# EspH is a hypervirulence factor for *Mycobacterium marinum* and essential for the secretion of the ESX-1 substrates EspE and EspF

**DOI:** 10.1371/journal.ppat.1007247

**Published:** 2018-08-13

**Authors:** Trang H. Phan, Lisanne M. van Leeuwen, Coen Kuijl, Roy Ummels, Gunny van Stempvoort, Alba Rubio-Canalejas, Sander R. Piersma, Connie R. Jiménez, Astrid M. van der Sar, Edith N. G. Houben, Wilbert Bitter

**Affiliations:** 1 Section Molecular Microbiology, Amsterdam Institute of Molecules, Medicines & Systems, Vrije Universiteit Amsterdam, Amsterdam, The Netherlands; 2 Department of Medical Microbiology and Infection Control, Amsterdam University Medical Centers, Amsterdam, the Netherlands; 3 Department of Medical Oncology, OncoProteomics Laboratory, Amsterdam University Medical Centers, Amsterdam, the Netherlands; University of Massachusetts Medical School, UNITED STATES

## Abstract

The pathogen *Mycobacterium tuberculosis* employs a range of ESX-1 substrates to manipulate the host and build a successful infection. Although the importance of ESX-1 secretion in virulence is well established, the characterization of its individual components and the role of individual substrates is far from complete. Here, we describe the functional characterization of the *Mycobacterium marinum* accessory ESX-1 proteins EccA_1_, EspG_1_ and EspH, *i*.*e*. proteins that are neither substrates nor structural components. Proteomic analysis revealed that EspG_1_ is crucial for ESX-1 secretion, since all detectable ESX-1 substrates were absent from the cell surface and culture supernatant in an *espG*_*1*_ mutant. Deletion of *eccA*_1_ resulted in minor secretion defects, but interestingly, the severity of these secretion defects was dependent on the culture conditions. Finally, *espH* deletion showed a partial secretion defect; whereas several ESX-1 substrates were secreted in normal amounts, secretion of EsxA and EsxB was diminished and secretion of EspE and EspF was fully blocked. Interaction studies showed that EspH binds EspE and therefore could function as a specific chaperone for this substrate. Despite the observed differences in secretion, hemolytic activity was lost in all *M*. *marinum* mutants, implying that hemolytic activity is not strictly correlated with EsxA secretion. Surprisingly, while EspH is essential for successful infection of phagocytic host cells, deletion of *espH* resulted in a significantly increased virulence phenotype in zebrafish larvae, linked to poor granuloma formation and extracellular outgrowth. Together, these data show that different sets of ESX-1 substrates play different roles at various steps of the infection cycle of *M*. *marinum*.

## Author summary

*M*. *tuberculosis* is a facultative intracellular pathogen that has an intimate relationship with host macrophages. Proteins secreted by the ESX-1 secretion system play an important role in this interaction, for instance by orchestrating the escape from the phagosome into the cytosol of the macrophage. However, the exact role of the ESX-1 substrates is unknown, due to their complicated interdependency for secretion. Here, we study the function of ESX-1 accessory proteins EccA_1_, EspG_1_ and EspH in ESX-1 secretion in *Mycobacterium marium*, the causative agent of fish tuberculosis. We found that these proteins affect the secretion of different substrate classes, which offers an approach to study the roles of these substrate groups. An *espG*_*1*_ deletion broadly aborts ESX-1 secretion and thus resulted in severe attenuation in a zebrafish model for tuberculosis, whereas EccA_1_ is only crucial under specific growth conditions. The most surprising results were obtained for EspH. This protein seems to function as a molecular chaperone for EspE and is as such involved in the secretion of a small subset of ESX-1 substrates. Disruption of *espH* showed a dual character: whereas this gene is essential for the successful infection of macrophages, deletion of *espH* resulted in significantly increased virulence in zebrafish larvae. These data convincingly show that different subsets of ESX-1 substrates play different roles at various steps in the mycobacterial infection cycle.

## Introduction

*Mycobacterium tuberculosis*, the etiological agent for the disease tuberculosis (TB), is still one of the most dangerous pathogens for global health [1]. Successful infection requires secretion of multiple virulence factors, facilitated by type VII secretion systems (T7SS). Pathogenic mycobacteria have up to five T7SS, called ESX-1 to ESX-5 [2], of which at least three are essential for growth and/or virulence [3,4]. The ESX-1 locus was the first T7SS to be identified. The loss of ESX-1 function in *Mycobacterium bovis* BCG is considered a decisive factor of attenuation of this vaccine strain [5]. Mouse infection experiments utilizing *M*. *tuberculosis* with a partial deletion in ESX-1 showed reduced granuloma formation, the characteristic pathological hallmark of mycobacterial disease [6,7]. Similarly, efficient granuloma formation, dissemination of disease and invasion of endothelial cells in the fish-pathogen *Mycobacterium marinum* is dependent on a functional ESX-1 secretion system [8–10]. More detailed analysis showed that ESX-1 substrates are required for phagosomal membrane rupture [11,12].

Thus far, about a dozen different proteins have been identified to be secreted through ESX-1, which can be divided in three subgroups, the Esx proteins, the PE/PPE proteins and the Esp proteins. Of these substrates, the Esp proteins are ESX-1 specific [13]. The ESX-1 substrates EsxA (ESAT-6) and EsxB (CFP-10) are secreted as an antiparallel heterodimer [14]. Interestingly, the limited structural data available for PE and PPE proteins also show that these proteins form a heterodimer [15–17]. These heterodimers form a four-helix bundle and contain a YxxxD/E secretion motif directly after the helix-turn-helix on one of the Esx proteins and on the PE protein [15,18]. The ESX-1 substrate EspB forms a similar four helix bundle with the conserved secretion motif at the same position in the structure and therefore does not seem to require a partner protein [17,19]. EsxA and EsxB are most intensively investigated of the different ESX-1 substrates [11,20–22] and EsxA is thought to be responsible for ESX-1 related virulence determinants [11,21–24]. EspA and EspB have additionally been implicated to be important for virulence [25,26]. However, studying the exact role of each substrate is complicated, as deletion of *esxA*/*esxB* abolishes secretion of all different Esp proteins [8,27], while *espA* and *espB* deletion mutants are unable to secrete EsxA/EsxB [25,27].

The ESX-1 secretion system consists of a membrane complex composed of the ESX conserved components (Ecc) EccB_1_, EccCab_1_, EccD_1_ and EccE_1_ [28,29], which is stabilized by the MycP_1_ protein [29]. The ESX-1 secretion system additionally contains the cytosolic accessory components EspG_1_ and EccA_1_. EspG functions as a specific chaperone of cognate PE/PPE substrates [30,31] and deletion of *espG*_*1*_ leads to a block in the secretion of PE35/PPE68_1 in *M*. *marinum* [31]. Loss of EspG_1_ in *M*. *tuberculosis* caused severe attenuation, both in cell infection and in mice [32]. EccA_1_ is a cytosolic AAA+ ATPase (ATPases Associated with diverse cellular Activities), which is essential for the EsxA secretion in both *M*. *tuberculosis* and *M*. *marinum* [33,34]. The *M*. *marinum eccA*_*1*_-null strain has been shown to be attenuated in zebrafish larvae [34]. However, its exact function is not further characterized.

In the *M*. *marinum*, the genes *espG*_*1*_ *(MMAR_5441)* and *eccA*_*1*_ *(MMAR_5443)* are separated in the *esx-1* locus by *espH (MMAR_5442)*. EspH-like proteins are unique for the ESX-1 system. EspD is a homologue of EspH, sharing 55% sequence identity in *M*. *tuberculosis*. EspD is encoded by the *espACD* locus, located more than 260 kb upstream of the ESX-1 gene cluster. Interestingly, *M*. *tuberculosis* EspD has a role in stabilizing the intracellular levels of the secreted substrate dimer EspA/EspC [35]. These observations suggest that EspH might function as a molecular chaperone.

Here, we study the role of three accessory proteins EspG_1_, EccA_1_ and EspH in *M*. *marinum* and could show that mutants in the corresponding genes displayed distinctive and contrasting virulence phenotypes, demonstrating that ESX-1 substrates play different roles in virulence. We additionally identified several potential new ESX-1 substrates.

## Results

### Individual ESX-1 components EspG_1_, EspH and EccA_1_, display distinctive effects on the secretion of ESX-1 dependent substrates

To study the role of accessory ESX-1 proteins EspG_1_, EccA_1_, and EspH in secretion, we created targeted knocked-out strains for *espH* and *eccA*_*1*_ and used the previously described *espG*_*1*_ knockout in *M*. *marinum* [31]. Deletion of the individual genes had no effect on bacterial growth in 7H9 medium (S1A Fig). However, colonies of the *eccA*_*1*_ mutant appeared dry with a rough-surface, while no phenotypic change was observed for the *ΔespG*_*1*_ and *ΔespH* colonies. In addition, qRT-PCR on total RNA extractions showed that the different deletions had no polar effect on the transcription of neighboring genes (S1B Fig).

**Fig 1:**
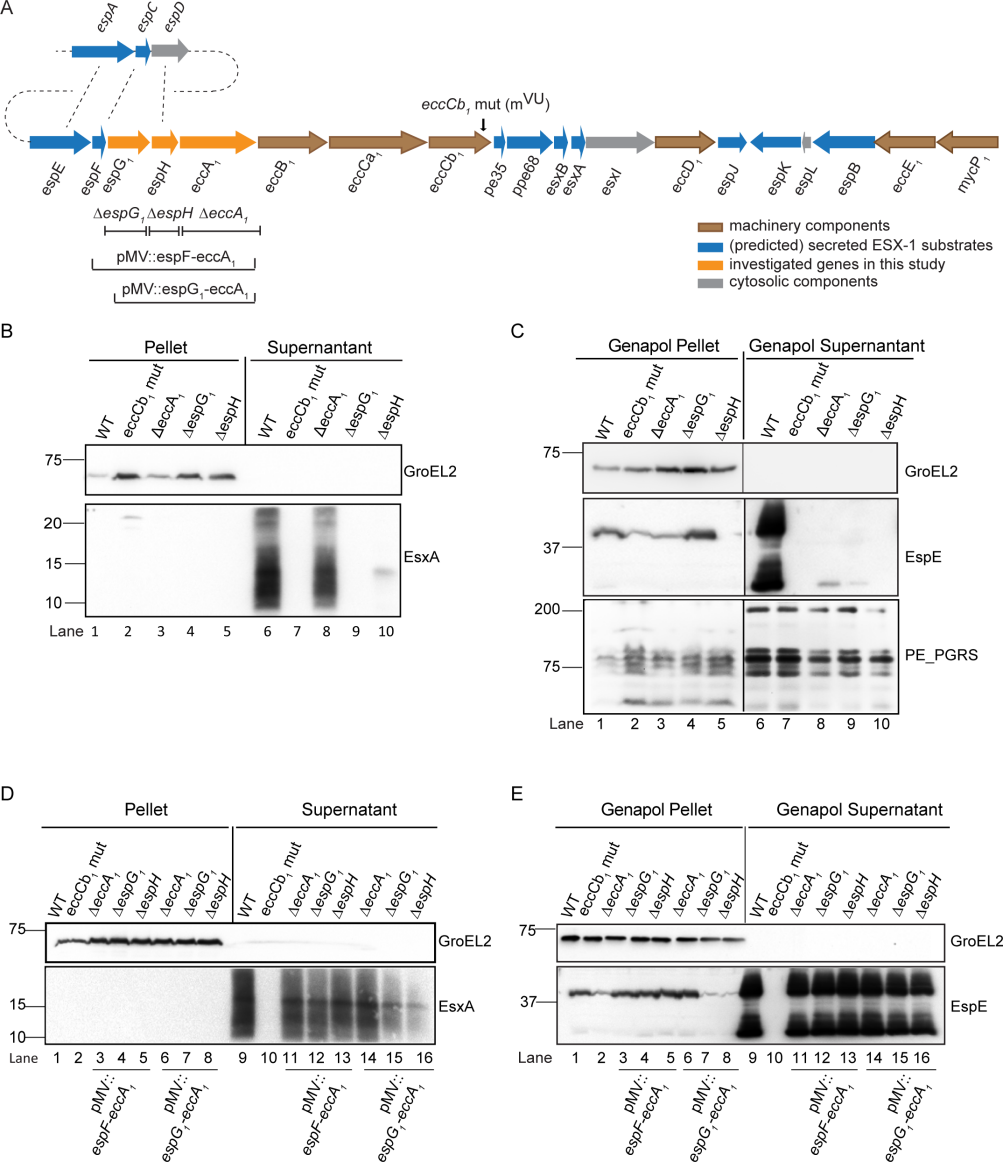
Mutants affected in the ESX-1 accessory proteins EspG_1_, EspH and EccA_1_ differently affect the ESX-1 secretome. **A.** Genetic organization of *espG*_*1*_-*espH*-*eccA*_*1*_ in the *esx-1* locus. Genes are color-coded according to the localization of their proteins—see key. **B and C.** Secretion analysis of EsxA and EspE substrates reveals that single deletion of *espG*_*1*_, *espH* and *eccA*_*1*_ affects secretion at different levels. Immunoblot analysis using protein preparations of wild-type *M*. *marinum* and the indicated mutants. In B we analyzed cell pellets not treated with detergent Genapol X-080 and culture supernatant fractions. In C we analyzed cell pellets treated with Genapol X-080 and the concomitant supernatant fractions. **D and E.** Complementation of the mutant strains fully restores ESX-1 secretion. In D the secretion of EsxA was analyzed and in E the secretion of EspE. In both experiments, GroEL2 was used as loading control and PE_PGRS as cell-surface control fraction. Equivalent OD units were loaded; 0.2 OD for pellet or Genapol pellet and 0.5 OD for supernatant or Genapol supernatant fractions.

Next, secretion analysis was performed using immunoblotting and a set of antibodies directed against known ESX-1 substrates. GroEL2 was included as a loading and lysis control. As a known ESX-1 negative mutant we included the M^vu^ strain, which has a frameshift mutation in *eccCb*_*1*_ [4,36] (Fig 1B, lane 6 and lane 7, respectively). Our analysis showed that EsxA was no longer secreted in the Δ*espG*_*1*_ strain (Fig 1B, lane 9), similarly as observed in a previous study from our group [31], but in contrast to the results obtained in *M*. *tuberculosis* [33]. Interestingly, the deletion of *espH* also resulted in a dramatic decrease in the secretion of EsxA (Fig 1B, lane 10). Surprisingly, and in contrast to what has been published previously [8,34], we observed that secretion of EsxA was reduced in the *eccA*_*1*_ mutant, but not completely aborted (Fig 1B, lane 8).

Next, we analyzed another ESX-1 substrate EspE (MMAR_5439), a highly abundant cell surface protein of *M*. *marinum*, which can be extracted from the cell surface using the mild detergent Genapol X-080 [37]. The surface localization of the ESX-5 dependent PE_PGRS proteins was included as controls. In the WT strain, EspE was secreted in two forms: a full-length protein of ~ 40 kDa and a putatively processed form of ~ 25 kDa (Fig 1C, lane 6). Surface localization of EspE was abolished in all the mutant strains (Fig 1C, lane 7 to lane 10). Notably, while EspE accumulated in the cell pellet of all non-secreting strains, this protein was not detected in the pellet fraction of the *espH* mutant (Fig 1C, lane 5), indicating that secretion of EspE was blocked at a different stage as compared to the other mutants.

To confirm that the observed secretion defects were caused by the targeted mutations, complementation plasmids were constructed. Two different complementation plasmids were used: the first one includes the genomic region from *espF (MMAR_5440)* to *eccA*_*1*_ *(MMAR_5443)*, whereas in the second plasmid only the *espG*_*1*_*-espH-eccA*_*1*_ locus was present. Complementing the knockout strains with either of these plasmids fully restored the secretion of EsxA and EspE in all of the mutants (Fig 1D and 1E).

### The absence of *eccA*_*1*_ causes a loss of EsxA secretion under specific growth conditions

**Fig 2:**
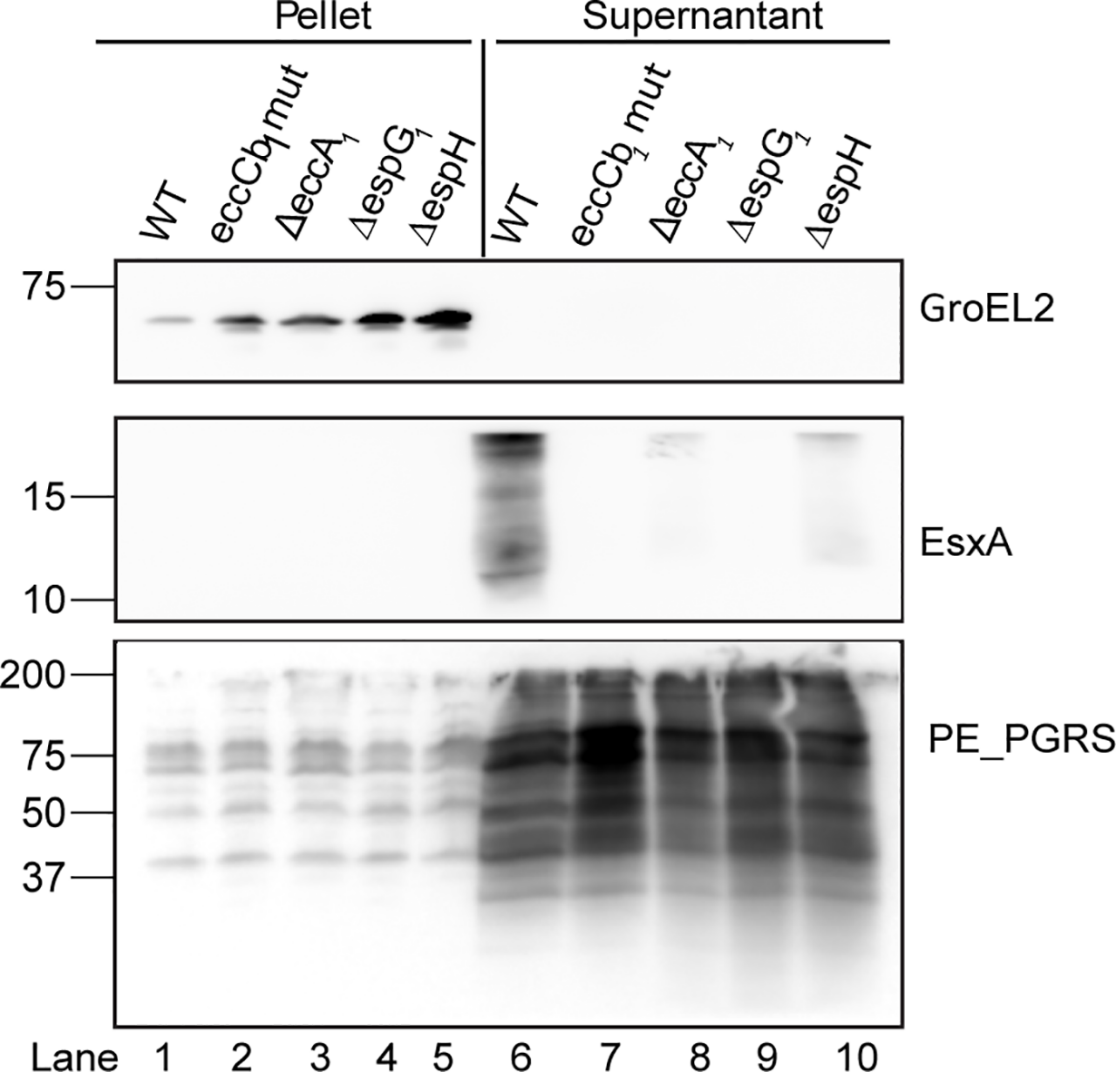
Secretion of EsxA by the *eccA*_*1*_ mutant is growth-medium dependent. Secretion analysis of the WT *M*. *marinum* M^USA^, the *eccCb*_*1*_ mutant and the knockout strains *espG*_*1*_, *espH* and *eccA*_*1*_ grown in Sauton’s defined medium. Immunoblot analysis with anti-EsxA confirmed a requirement of EccA_1_ for a full secretion of EsxA when cells were grown in this medium. Anti-GroEL2 was used as a loading and lysis control for all samples. Anti-PGRS antibodies, staining the ESX-5 dependent substrates PE_PGRS proteins, were used as a supernatant control for all samples. Equivalent OD units were loaded; 0.3 OD for pellet and 0.6 OD for supernatant or supernatant fractions.

A major discrepancy with previous publications was our finding that EccA_1_ has a limited effect on EsxA secretion. Previously, Gao *et al*. showed, using the same *M*. *marinum* background strain, that EccA_1_ is crucial for ESX-1 secretion [8,34]. We realized that there is a difference in the growth conditions between the two studies; we used 7H9 medium whereas Gao *et al*. used Sauton medium [8,34]. To test whether the observed differences could be linked to a difference in growth condition, secretion analysis was performed on cultures grown in Sauton medium. Interestingly, whereas the results for Δ*espG*_*1*_ and Δ*espH* were identical (Fig 2, lane 9 and lane 10, respectively), EsxA was no longer secreted in the *eccA*_*1*_ mutant strain (Fig 2, lane 8), which shows that the role of EccA_1_ in EsxA secretion is dependent on the growth condition.

### Secretome analysis of accessory ESX-1 protein mutants by LC-MS/MS

The proteome of a number of ESX-1 targeted knockout strains of *M*. *marinum* has been determined previously [38]. However, this study did not include an *espH* mutant and the cell surface proteome was not analyzed. In order to obtain a comprehensive and detailed view, the complete secretomes of our mutant strains, the corresponding complemented strains and both the WT and ESX-1 secretion mutant *eccCb*_*1*_ were analyzed by mass spectrometry. As some ESX-1 substrates are efficiently secreted into the culture supernatant, while others mainly remain attached to the cell surface [37], cells were grown with or without Tween 80 to study secreted proteins in the medium or the cell surface proteins, respectively. The cell surface proteins were extracted from the bacterial cells using Genapol X-080.

**Fig 3:**
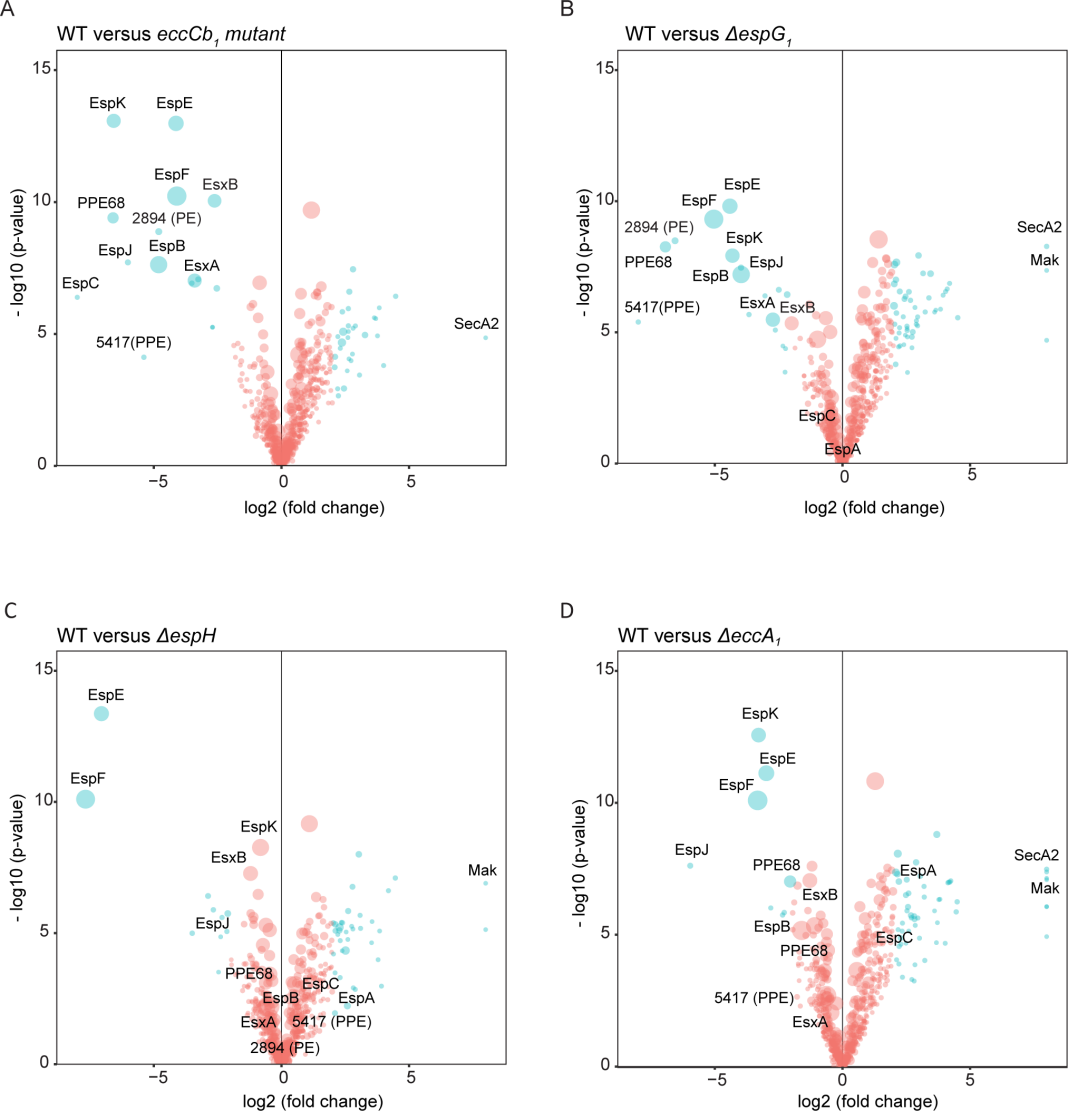
Quantitative proteomics analysis of the Genapol-enriched fractions of different *M*. *marinum* ESX-1 mutant strains. Volcano plots representing the statistical significance of changes of cell-surface enriched proteins between the WT *M*. *marinum* and each ESX-1 mutant. The vertical lines depict p value on the–log base 10 scale. The horizontal lines denote fold change on the log base 2 scale. Only proteins with an accumulative number of more than 10 spectral counts are shown. Each dot corresponds to a single identified protein and the size of the dots correlates to the accumulative spectral counts of the protein of the WT and the corresponding mutant. Proteins with a spectral count difference of more than eight folds were set to eight. In blue: proteins that showed more than 4 folds of change, otherwise in red. Only putative ESX-1 substrates, SecA2 and Mak are annotated. **A**. WT versus the *eccCb*_*1*_ mutant. **B**. WT versus the *ΔespG*_*1*_ mutant. **C**. WT versus the *ΔespH* mutant. **D.** WT versus the *ΔeccA*_*1*_ mutant.

**Fig 4:**
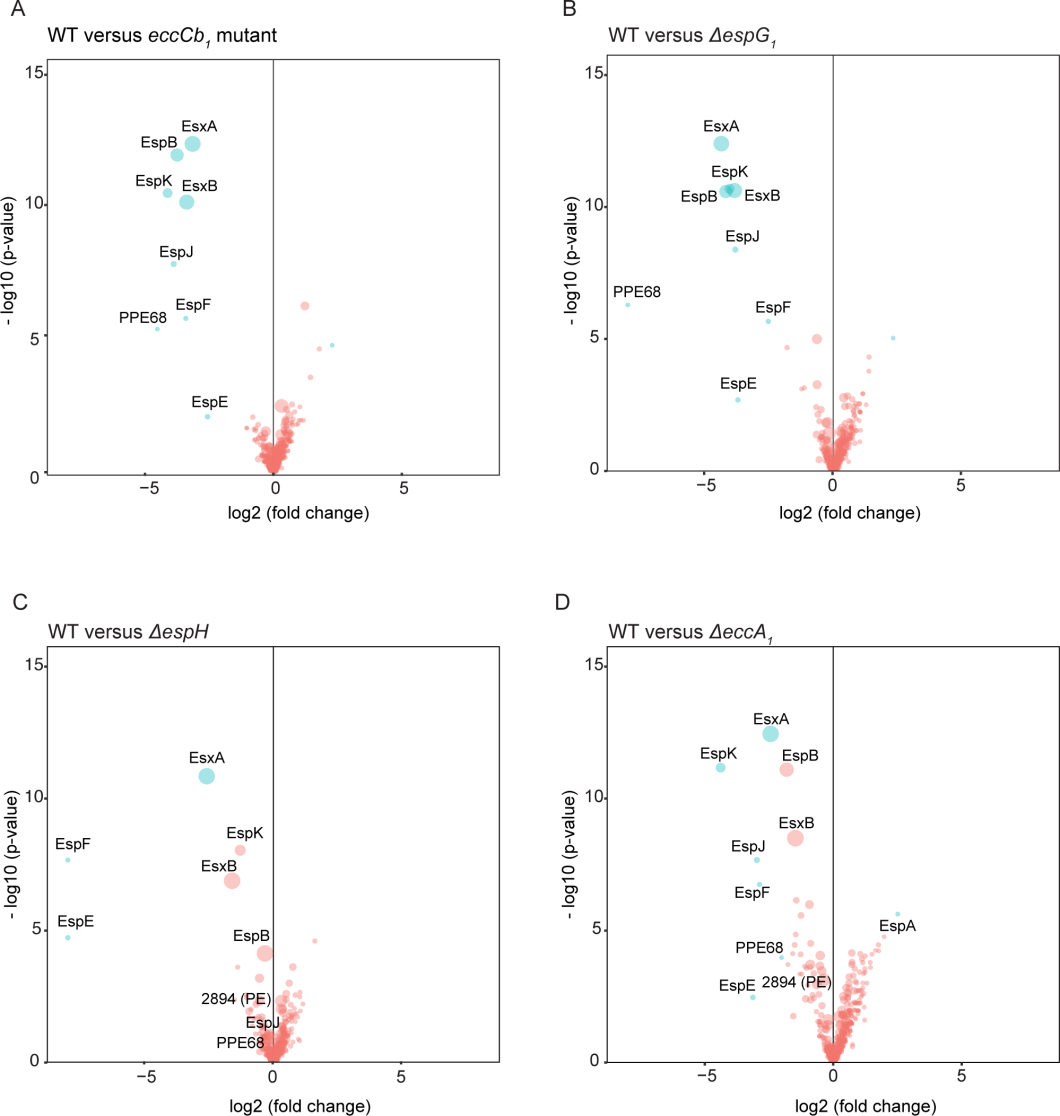
Quantitative proteomics analysis of the supernatant of different *M*. *marinum* ESX-1 mutant strains. Volcano plots representing the statistical significance of changes of the secreted proteins in the supernatant between the WT *M*. *marinum* and each ESX-1 mutant. The same quantitative method was used as in Fig 3 for the Genapol-enriched fractions. **A**. WT versus the *eccCb*_*1*_ mutant. **B.** WT versus the *ΔespG*_*1*_ mutant. **C.** WT versus the *ΔespH* mutant. **D.** WT versus the *ΔeccA*_*1*_ mutant.

For the ESX-1 secretion (*eccCb*_*1*_) mutant, a massive reduction in the secretion of all known ESX-1 substrates, i.e. EsxA (MMAR_5449), EsxB (MMAR_5450), EspB (MMAR_5457), EspC (MMAR_4167), EspE (MMAR_5439), EspF (MMAR_5440), EspJ (MMAR_5453), EspK (MMAR_5455) and PPE68 (MMAR_5448), was observed, both in the cell surface-enriched fractions (Fig 3A) and the culture supernatants (Fig 4A). These results are in line with published data [38]. Also the secretion of several other proteins, including the PE protein MMAR_2894 and PPE protein MMAR_5417, was blocked, suggesting they are novel ESX-1 substrates. This notion is strengthened by the fact that these two proteins are homologous to the PE and PPE protein encoded by the *esx-1* locus. For the other proteins that showed reduced spectral counts in the cell surface fractions it is more difficult to draw any conclusion. First of all, the difference in secretion levels are smaller as compared to the known ESX-1 substrates (Fig 3), but furthermore they lack known characteristics of T7SS substrates, such as the YxxxD/E secretion motif preceded by a predicted helix-turn-helix structure. The *espG*_*1*_ mutant showed similar secretion profiles as the *eccCb*_*1*_ mutant (Fig 3B and Fig 4B), although the secretion of EspB, EspK and EspE seemed to be slightly less severely affected. This suggests that EspG_1_ is not only required as a chaperone for its cognate PE/PPE substrates, but plays a more central role in the secretion of all ESX-1 substrates. The secretion of all ESX-1 substrates returned to WT levels in the *espG*_*1*_ mutant carrying the pMV361::*espF-eccA*_*1*_ complementation plasmid (S2A and S2B Fig).

The secretome profiles of the *eccA*_*1*_ mutant in 7H9 medium showed only a mild reduction of ESX-1 substrates in both cell surface and supernatant fractions (Fig 3D and Fig 4D). For instance, EsxA and EsxB secretion was five and two-fold decreased, respectively, while in the *eccCb*_*1*_ mutant the reduction of both was 10 fold (Fig 4D). The substrates EspE, EspF, EspJ and EspK are more affected by the *eccA*_*1*_ mutation than the other substrates in both protein fractions. In concordance with the data obtained by immunoblotting, the complementation of the *eccA*_*1*_ mutant with pMV361::*espF-eccA*_*1*_ plasmid restored the secretion of all ESX-1 substrates (S2A and S2B Fig).

Deletion of *espH* resulted in a severe reduction of EspE and EspF (Fig 3C), in line with our immunoblot analysis. This reduction was in fact almost complete, both in the fraction of the surface proteins (determined LC-MS/MS) and in the bacterial pellet (determined by immunoblotting), which again suggests instability of intracellular EspE/EspF in the absence of EspH. This effect was restored when the complementation plasmid was introduced (S2A and S2B Fig). Interestingly, the effects of the *espH* deletion on secretion of EsxA and EsxB was only mild as compared to the *eccCb*_*1*_ mutant, while the effects on other ESX-1 substrates, such as EspB, EspK and EspJ were also only minor (Fig 4C). This indicates that *ΔespH* has a specific secretion defect for a subset of ESX-1 substrates and there is no substrate dependency between EspE/EspF and other Esp proteins.

Surprisingly, we also identified some proteins that were present in significantly increased amounts in the cell surface enriched fractions of various mutants. One of these proteins is SecA2, a cytosolic component of the Sec transport system and proposed to contribute to the virulence of *M*. *tuberculosis and M*. *marinum* [39,40]. SecA2 was present in higher amounts in all mutants except the Δ*espH*, suggesting a link with intracellular accumulation of EspE/EspF. Another intriguing observation is an increase of Mak in the Δ*espG*_1_, Δ*espH* and the Δ*eccA*_*1*_ (Fig 3B, 3C and 3D, respectively). Mak is a mycobacterial maltokinase whose function is involved in the glycan synthesis from trehalose [41] and considered to be essential for the growth of *M*. *tuberculosis* [42]. This could suggest that there is an indirect effect of ESX-1 secretion on the synthesis of the mycobacterial capsule.

### EspE specifically interacts with EspH in *M*. *marinum*

The observation that EspH mainly affects the secretion of EspE/EspF and that EspE could not be detected in the *espH* mutant pellet fraction raised the hypothesis that EspH could either regulate the transcription of *espE/espF* or stabilize EspE/EspF at the protein level. To get more information on the putative function of EspH we used the protein structure prediction program Phyre2 [43]. This analysis showed that part of EspH (region between amino acid 65 and 135) is predicted to share structural similarity to YbaB proteins of *Escherichia coli* and *Haemophilus influenza*. Although the sequence identity with these proteins is low (15%) the confidence of the structural homology is very high (97%). Because YbaB is reported to be a small DNA-binding protein that plays a regulatory role [44], an effect on transcription regulation could be possible. Therefore, we measured the effect of *espH* deletion on *espE* and *espF* mRNA levels. Because the EsxA secretion was reduced in the *espH* mutant, *esxA* mRNA level was checked as well. Total mRNA was extracted from the WT M^USA^, *eccCb*_*1*_ mutant and the Δ*espH* strain, and qRT-PCR was performed using primer sets for *espE*, *espF* and *esxA*. The results showed that the mRNA levels of all three genes were comparable to those of the *eccCb*_*1*_ mutant strain analyzed (S3A Fig). Thus, we could disprove the possibility that EspH regulates *espE* at the transcriptional level.

Next, we studied a direct interaction of EspH with EspE and/or EspF. Based on the high homology of EspE with EspA and EspF with EspC, we speculated that, similarly to EspC/EspA [45], EspF might be secreted together with EspE. We therefore constructed a plasmid containing *espE*/*espF* in which *espE* was modified to express a C-terminal Strep tag. We also introduced a His tag at the C terminus of EspH in the *espG*_*1*_/*espH*/*eccA*_*1*_ complementation plasmid. Introduction of both plasmids in the WT and Δ*espH* mutant resulted in surface localized EspE, as judged by immunoblot analysis of the cell surface extracted protein preparations (S3B Fig). These results show that the addition of the Strep tag to the C terminus of EspE and the His-tag to EspH did not affect the functionality of these proteins in the secretion process.

**Fig 5:**
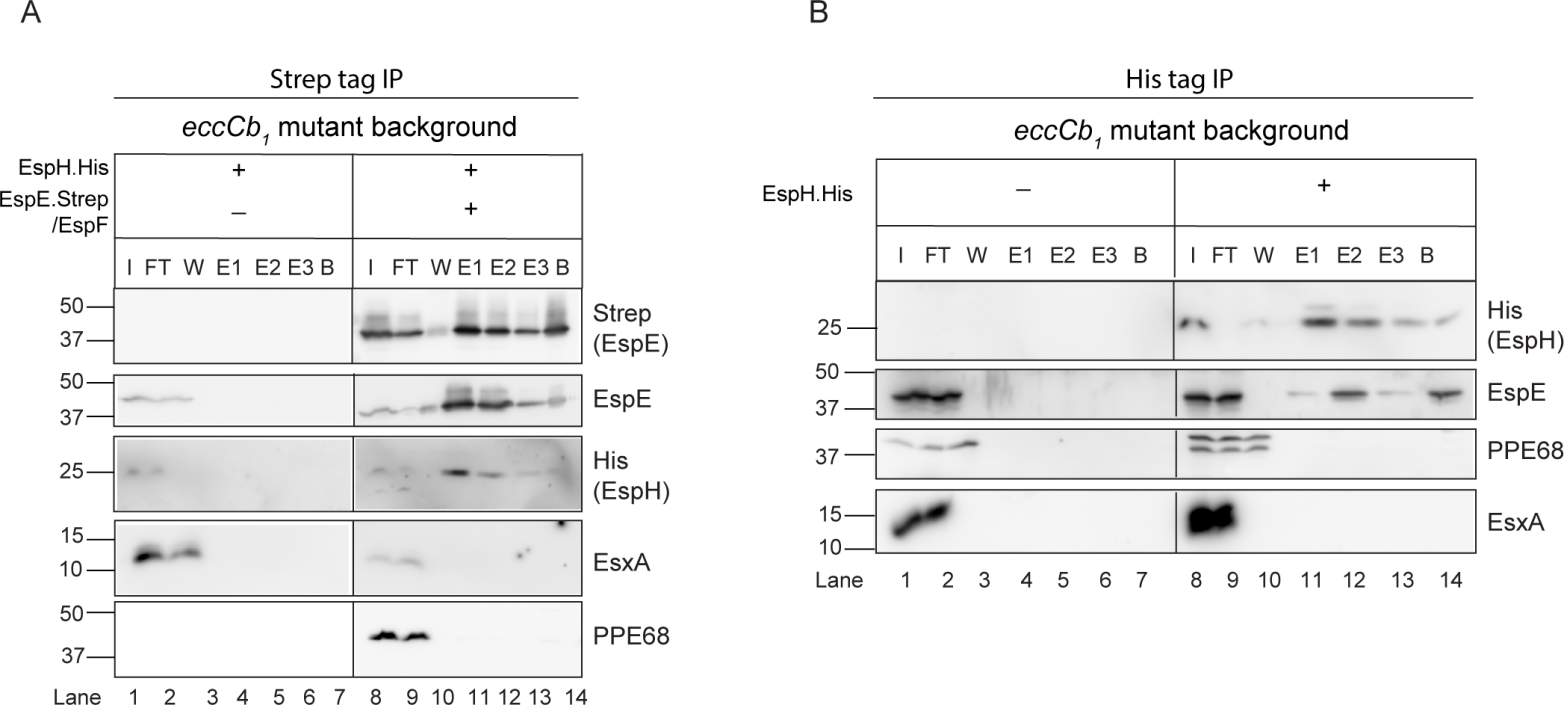
EspH specifically interacts with EspE in *M*. *marinum*. **A.** Immunoblots of pulldown assays using Strep-tactin agarose. EspE-Strep was purified from soluble lysates of the *eccCb*_*1*_ mutant expressing only EspE-Strep/EspF or EspE-Strep/EspF together with EspH-His. A strain containing empty plasmids was included as negative control. Total input material (I), unbound proteins (FT), the final washing step (W), three fractions of eluted proteins (E1, E2, E3) and boiled beads fractions were separated by SDS-PAGE and further immunoblotted using antisera directed against the Strep- or His-tag. The elution fractions were loaded 10 times more compared to the other fractions. Endogenous EspE, PPE68 and EsxA substrates were detected using anti-EspE, anti-PPE68 and anti-EsxA, respectively. **B.** Immunoblots of pulldown assays using Ni-NTA beads. EspH-His proteins were purified from soluble lysates of the *eccCb*_*1*_ strain carrying a plasmid expressing EspH-His or the corresponding empty plasmid. Total input material (I), unbound proteins (FT), the last washing step (W), proteins eluted with 50 mM (*E1*), 100 mM (*E2*), and 200 mM (*E3*) imidazole and boiled bead fraction were separated by SDS-PAGE and probed with His-specific antiserum. The elution fractions were loaded 10 times more compared to the other fractions. Endogenous EspE, PPE68 and EsxA proteins were detected using anti-EspE, anti-PPE68 and anti-EsxA, respectively.

To study the interaction of EspE and EspH, we overexpressed EspE-Strep/EspF and EspH-His in the *eccCb*_*1*_ mutant strain. The ESX-1 secretion system is defective in this strain and therefore EspE and EspH accumulate in the cytosol, which allows their analysis and co-purification. The subcellular localization of EspE and EspH was examined by a subcellular fractionation procedure, showing that EspE-Strep was partially soluble while EspH-His was exclusively present in the soluble fraction (S3C Fig). Next, we used StrepTactin beads to purify Strep-tagged EspE from these soluble fractions. Immunoblot analysis showed that EspE-Strep was efficiently purified. Importantly, EspH-His, appearing as a ~ 25 kDa band, was only present in the elution fractions when expressed in the presence of EspE-Strep (Fig 5A). In contrast, the ESX-1 substrates PPE68 and EsxA were not co-purified and both remained in the flow-through fraction.

To confirm this EspE-EspH interaction, a reciprocal pull-down assay was performed using Ni-NTA beads and lysates of the *eccCb*_*1*_ mutant containing EspE-strep/EspF only or EspE-strep/EspF and EspH-His. Immunoblot analysis confirmed the efficient purification of EspH-His (Fig 5B). Using anti-EspE on these samples showed co-elution of endogenous EspE only in the presence of the His-tagged EspH (Fig 5B). Again, PPE68 and EsxA were only found in the flow-through fraction, indicating that they do not bind EspH. In conclusion, these data confirmed that EspH specifically interacts with EspE in the cytosol of *M*. *marinum* and this interaction is probably required for EspE secretion.

### The *espH* mutant is attenuated in phagocytic cells and shows strongly reduced hemolysis

**Fig 6:**
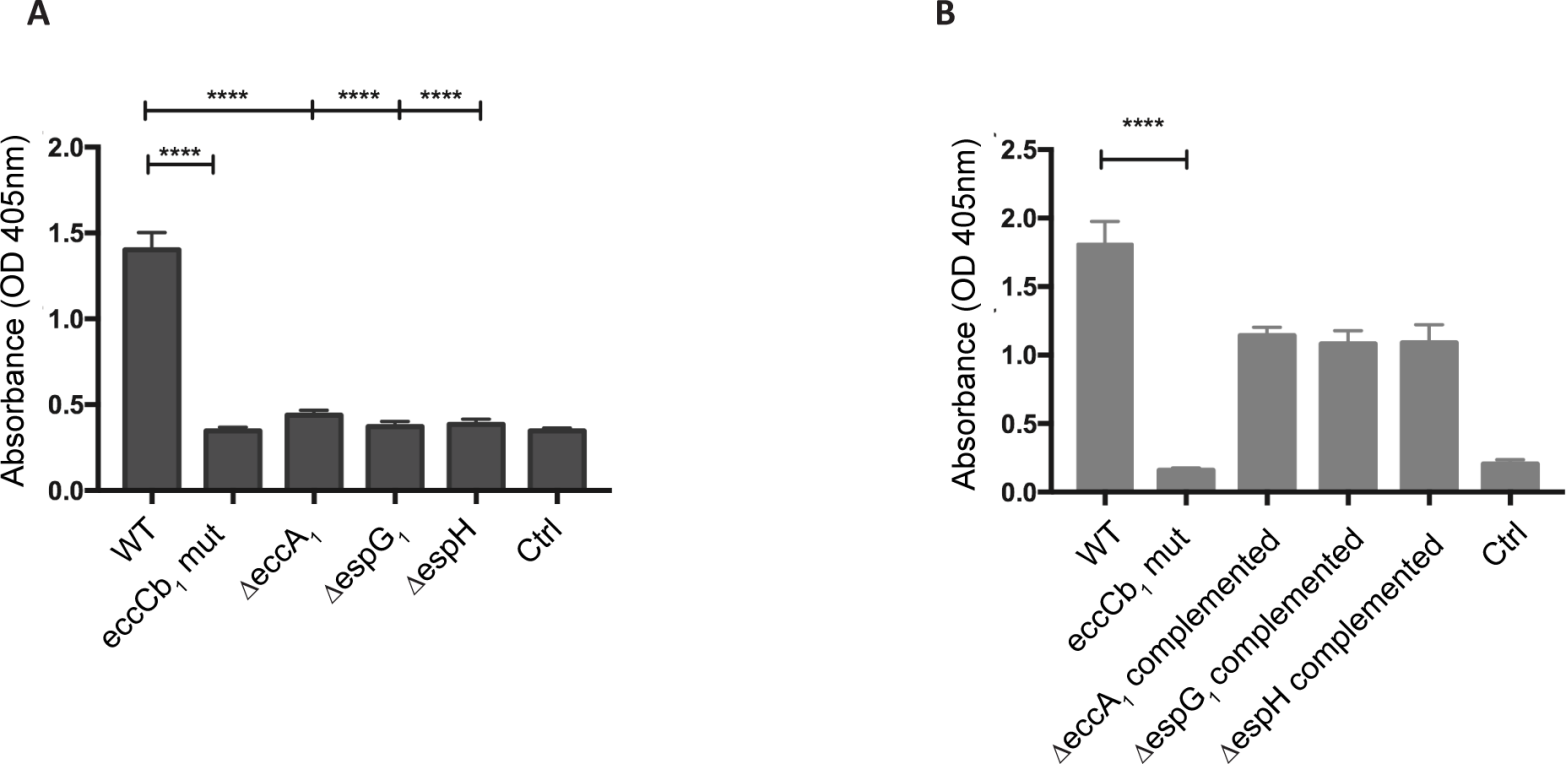
ESX-1 mutant strains have lost hemolytic activity. Contact-dependent hemolysis of red blood cells (RBCs) by various *M*. *marinum* strains grown in the presence of Tween-80. Hemolysis was quantified by determining the OD_405_ absorption of the released hemoglobin. The data shown here is generated from two independent experiments, each time in triplicates. In **A**, the ESX-1 mutants and in **B** the complemented strains with restored hemolytic activity are shown. Significance is indicated, **** < 0.0001. Ctrl = control sample with PBS.

ESX-1 functioning in *M*. *marinum* has been associated with lysis of red blood cells [8]. Because of this, the hemolysis assay has been employed as a model for the ESX-1-dependent lysis of (phagosomal) membranes [8]. Prior work suggested that the ESX-1 associated membrane lytic activity was mediated by EsxA through its pore-forming activity [21,46]. Because the deletion of *espG*_*1*_, *espH* and *eccA*_*1*_ differently affected the secretion of EsxA, we examined to what extend these mutant strains were able to disrupt erythrocytes. While we confirmed that our WT strain showed hemolysis (Fig 6A), both the *eccCb*_*1*_ and *ΔespG*_*1*_ mutant strain lost this ability, in line with the absence of ESX-1 substrates in the culture supernatant (Fig 6A). Interestingly, the Δ*espH* and Δ*eccA*_*1*_ strains were also non-hemolytic, although these strains were still able to secrete EsxA to significant levels (Fig 6A). The defects in hemolysis by the knockout strains were restored when the complemented plasmids were introduced into these mutant strains (Fig 6B). As in the Δ*espH* and Δ*eccA*_*1*_ mutants mainly the secretion of different Esp proteins are specifically affected, our findings indicate that not a single ESX-1 substrate, such as EsxA, but a combination of different Esp proteins, are responsible for the hemolytic phenotype.

**Fig 7:**
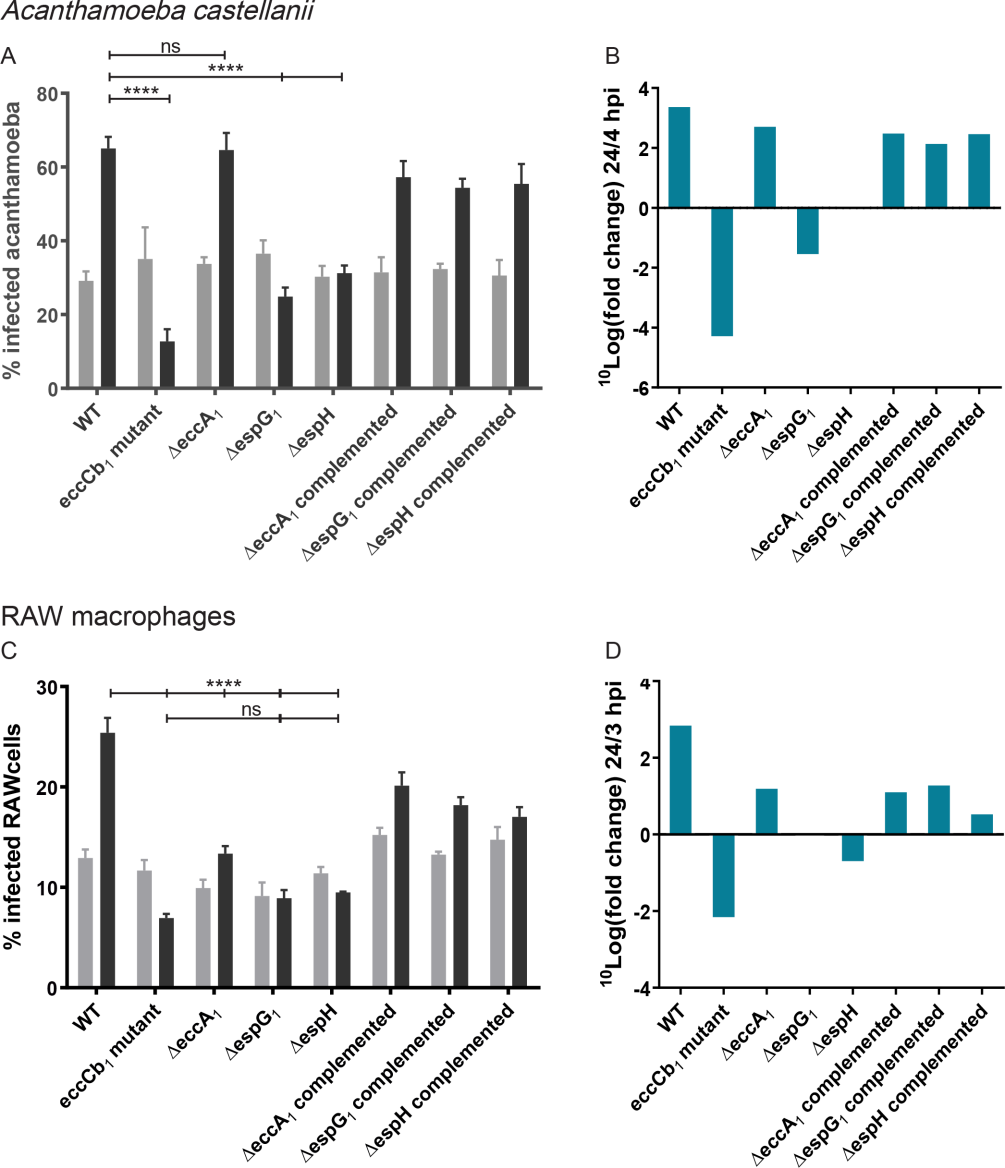
Intracellular growth of Δ*eccA*_*1*_, Δ*espG*_*1*_ and Δ*espH* in different hosts. **A.** Flow cytometry experiment showing percentage of infected *A*. *castellanii* at 4 hours post infection (hpi) versus 24 hpi, graph shows pooled data from two independent experiments. **B.** Graph shows fold change in percentage infected *A*. *castellanii* presented in A. **C.** Similar flow cytometry experiment with infected RAW macrophages when comparing percentage infected cells at 3hpi and 24 hpi, graph shows representative data of 1 out of 3 biological replicates.,. **D.** Graph shows fold change in percentage infected RAW macrophages presented in C. **** = p<0.001, ns = non-significant.

To further characterize the function of the different ESX-1 substrate subsets, we used different phagocytic cells to study the ability of the mutant strains to survive and replicate within these cells. Phagocytic cells from mice (RAW macrophage cell line) and the protozoa *Acanthamoeba castellanii* were infected with green fluorescent protein (GFP)-expressing mycobacteria and infection levels were quantified by flow cytometry at different time points. As shown before, the *eccCb*_*1*_ mutant was strongly attenuated in both *A*. *castellanii* and RAW cells (Fig 7; [47]), showing a 2-fold reduction in the number of infected cells after 24 h. As expected, based on the proteome profiles, the *ΔespG*_*1*_ strain showed an attenuated phenotype similar to the *eccCb*_*1*_ mutant. For the Δ*espH* mutant, the proportion of infected *A*. *castellanii* cells did not change over time (Fig 7B), while in RAW macrophages a slight reduction of infected cells at 24 hpi could be observed (Fig 7C, p = ns). Infection with the Δ*eccA*_*1*_ mutant resulted in an increase of infected cells over time, for both *A*. *castellanii* and RAW cells, and was therefore less attenuated as compared to the other mutants (Fig 7B and 7D). Although this strain was able to infect *A*. *castellanii* to the same extend as the WT strain, infection with this mutant was not as successful as WT infection in RAW macrophages (Fig 7A, ns; Fig 7C, p < 0.001).

Taken together, our data show the importance of *espG*_*1*_ in achieving successful infection of phagocytic cells, while the loss of *eccA*_*1*_ only marginally affects the ability of *M*. *marinum* to survive and replicate in a phagocytic host cell. These findings are in line with the proteomic analysis, *i*.*e*. the *espG*_*1*_ mutation has a strong effect on secretion of all ESX-1 substrates, while deleting *eccA*_*1*_ only results in a mild secretion defect. EspH, which seems to mainly influence EspE and EspF secretion, is also important for infecting phagocytes, but to a lesser extent than EspG_1_.

### *In vivo* virulence phenotype of *eccA*_*1*_ and *espG*_*1*_ mutant strains is similar to their *in vitro* phenotype

To study whether the individual ESX-1 proteins play a role during infection *in vivo*, we used the zebrafish larva-*M*. *marinum* infection model. Larvae were systemically infected with the fluorescently labeled mutant, complemented and WT strains and infection was analyzed 4-days post infection (dpi) by fluorescence microscopy. In addition, L-plastin staining was performed to visualize phagocytic cells in order to study the formation of early granulomas by confocal microscopy.

**Fig 8:**
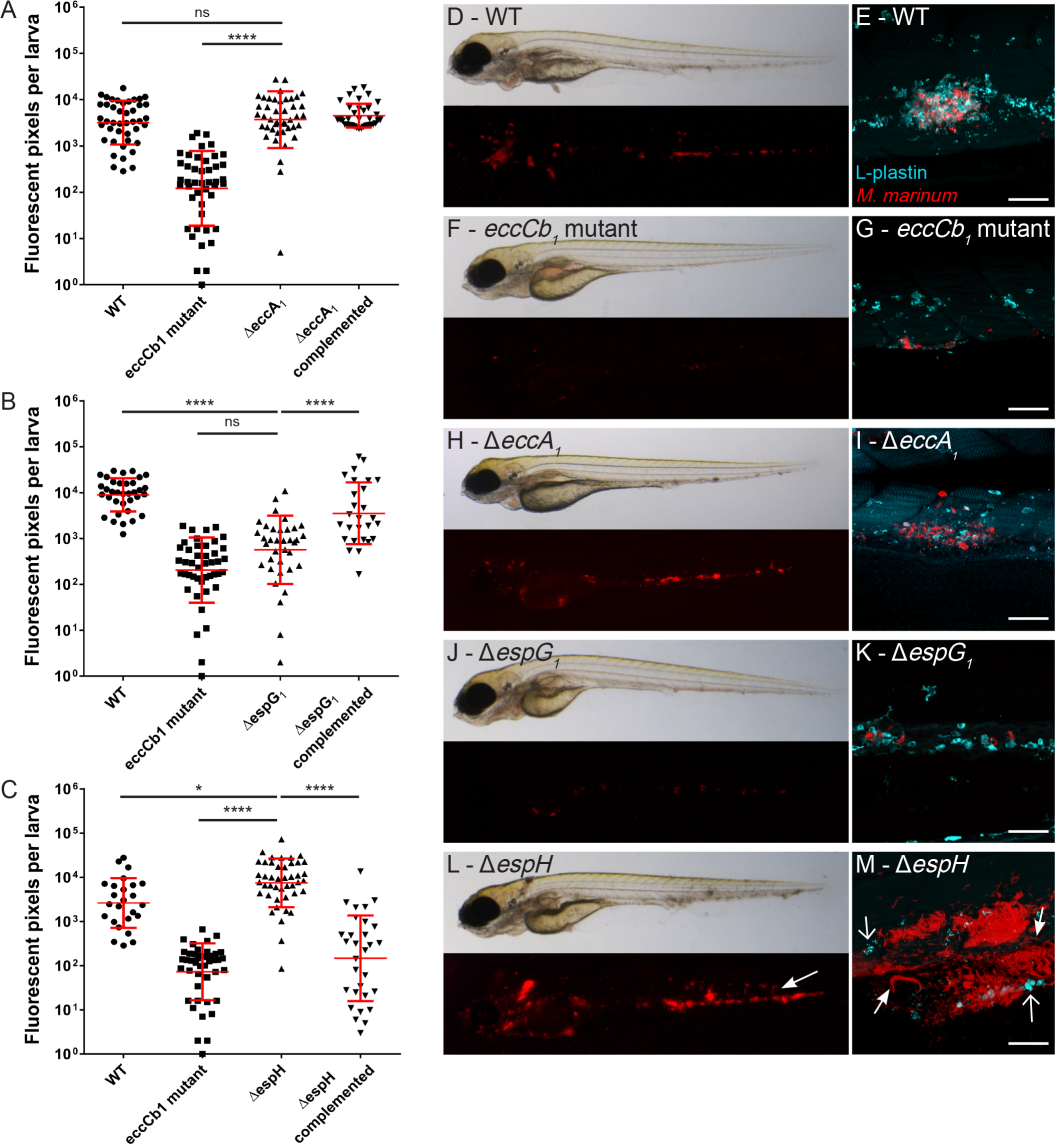
*In vivo* effect of Δ*eccA*_*1*_, Δ*espG*_*1*_ and Δ*espH* in zebrafish larvae. Graphs A-C show relative levels of infection as determined by automated pixel count software for infection of zebrafish larvae. The larvae were infected with ~75–150 CFU red fluorescent *M*. *marinum* mutant strains and analyzed at 4 dpi. Graphs show combined data of three independent biological replicates per mutant strain, each dot represents one larva. Bars represent mean and standard error of the mean. **A.** Systemic infection of zebrafish larvae with *M*. *marinum* Δ*eccA*_*1*_, **B.** *M*. *marinum* Δ*espG*_*1*_ and **C.** *M*. *marinum* Δ*espH*, * = <0.05, **** <0.001. Representative bright field and corresponding fluorescent images are depicted in: **D.** WT infection, **F.** *eccCb*_*1*_ mutant infection, **H.** *M*. *marinum* Δ*eccA*_*1*_, **J.** *M*. *marinum* Δ*espG*_*1*_, **L.** *M*. *marinum* Δ*espH*. Confocal imaging of a single cluster of infected L-plastin labeled phagocytic cells (cyan) in the tail of infected larvae confirmed the phenotype seen in fluorescent imaging: **E.** WT infection, **G.** *eccCb1* mutant infection, **I.** *M*. *marinum* Δ*eccA*_*1*_, **K.** *M*. *marinum* Δ*espG*_*1*_, **M.** *M*. *marinum* Δ*espH*, depicting a cording phenotype (closed arrows) and intense fluorescent spots suggestive for phagocytic cell debris (open arrows). Scale bar E, G, I, K, M = 50 μm.

Infection of zebrafish larvae with the Δ*espG*_*1*_ and Δ*eccA*_*1*_ mutant strains resulted in infection levels as expected from the previous experiments, *i*.*e*. the Δ*espG*_*1*_ showed a similar level of attenuation as the *eccCb*_*1*_ mutant, while the Δ*eccA*_*1*_ mutant infections were similar to wildtype infection (Fig 8A, 8D and 8H for Δ*eccA*_*1*_; Fig 8B, 8F and 8J for Δ*espG*_*1*_). Higher magnification of individual infection loci in Δ*eccA*_*1*_ infected larvae revealed recruitment of phagocytic cells and formation of early granulomas comparable to infection with WT (Fig 8E for WT, n = 12 larvae; Fig 8I for Δ*eccA*_*1*_, n = 8 larvae). In contrast, confocal imaging of Δ*espG*_*1*_ infected fish showed a predominance of single infected macrophages and formation of very small clusters of these infected macrophages similar to infection with the *eccCb*_*1*_ mutant (Fig 8G for *eccCb*_*1*_ mutant, n = 10 larvae; Fig 8K for *ΔespG*_*1*_, n = 7 larvae).

Together, this shows that *espG*_*1*_, but not *eccA*_*1*_, plays a major role in early stages of infection *in vivo*. Moreover, since these strains show a comparable behavior during *in vitro* and *in vivo* infections, this indicates functional similarities for these genes in protozoa, mouse macrophages and zebrafish larvae.

### The absence of *espH* results in a hypervirulent phenotype in zebrafish larvae

In contrast to the Δ*espG*_*1*_ and Δ*eccA*_*1*_ strain, the behavior of Δ*espH* in zebrafish larvae was completely different from its attenuated phenotype *in vitro*. Systemic infection of zebrafish larvae resulted in an increased bacterial load as compared to WT infection (Fig 8C; p < 0.05). Large bacterial clusters and a phenotype known as cording were seen in fluorescence images (Fig 8L, arrow), especially at higher magnification of individual clusters (Fig 8L, closed arrow, n = 15 larvae). Cording in zebrafish has been associated with extracellular growth [48]. In addition, very limited numbers of intact phagocytic cells and the presence of fluorescent spots suggestive for phagocytic cell debris were observed (Fig 8L, open arrow).

**Fig 9:**
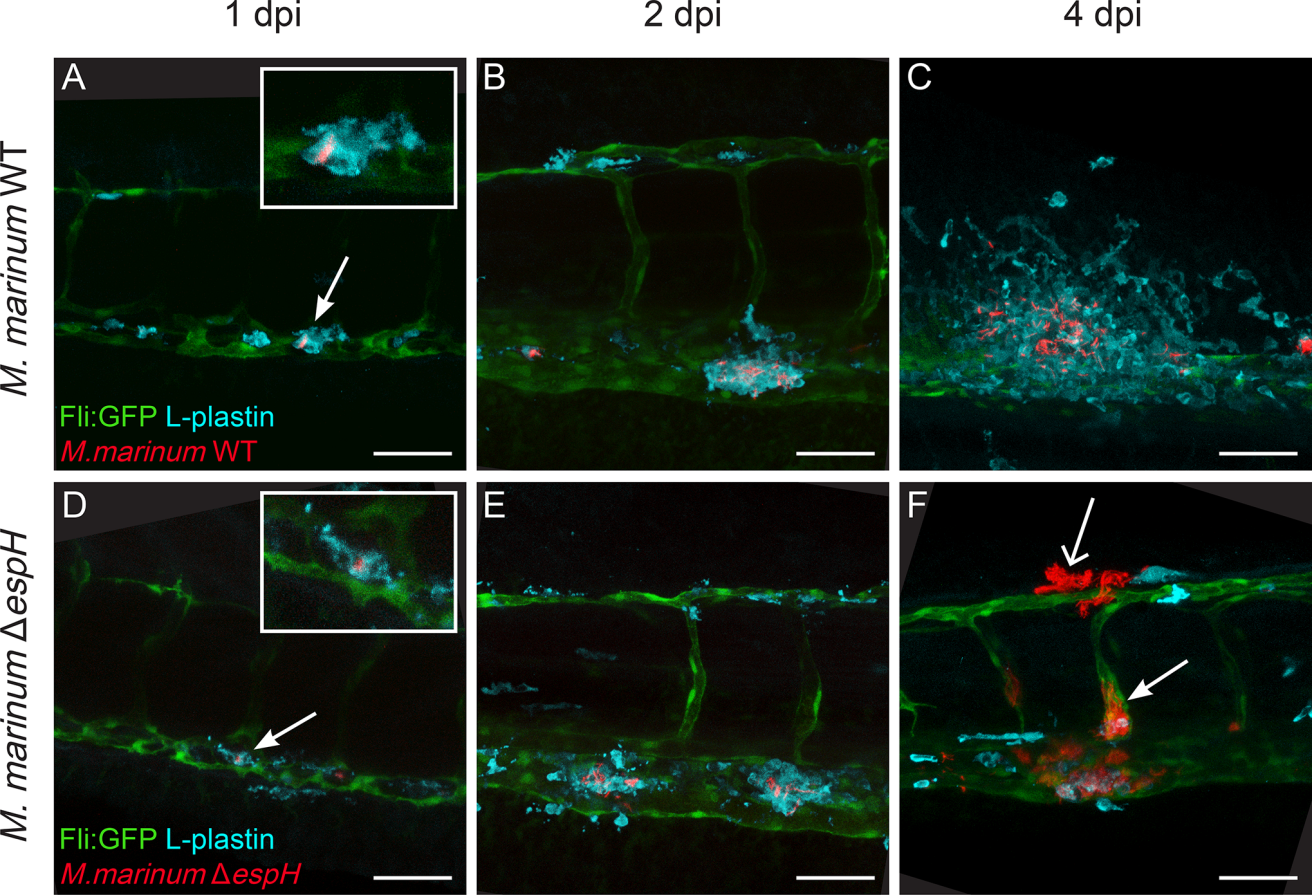
*EspH-mutant* strain is hypervirulent in zebrafish larvae. **A-C.** Systemic *M*. *marinum* WT infection (red) of Tg(fli:GFP) larvae with green fluorescent blood vessels was followed over time, representative images are shown in A. 1dpi, B. 2dpi, C. 4dpi. Larvae were stained with anti-L-plastin to label phagocytic cells (cyan). **D-F.** Representative images of systemic infection with *M*. *marinum* Δ*espH* over time in D. 1dpi, E. 2dpi, F. 4dpi. Scale bar = 50 μm.

These observations raised the question whether this phenotype is still preceded by granuloma formation or if this mutant strain is preventing early granuloma formation by inducing rapidly host cell death. Therefore, larvae were systemically infected with either Δ*espH* or WT *M*. *marinum* as control and monitored daily for 4 consecutive days (Fig 9). Mycobacteria were phagocytosed by L-plastin positive phagocytic cells at 1 dpi in both groups (Fig 9A and 9D). Subsequently, phagocytic cells were recruited and early granulomas started to form (Fig 9B and 9E). However, at 4 dpi, in larvae infected with the Δ*espH* strain a strong decrease in phagocytic cells and increase in bacterial growth was observed (Fig 9C and 9F). In the absence of phagocytic cells bacteria were able to show cording in both blood vessels (Fig 9F, closed arrow) and tissue (Fig 9F, open arrow).

Taken together, the Δ*espH* mutant seems to have a host-specific or *in vivo-*specific effect, illustrated by a hypervirulent phenotype seen in zebrafish larvae, but not in cell infections *in vitro*. Therefore, our data indicates that EspH is not required for initial phagocytosis, recruitment of cells and primary establishment of early granulomas, but this protein, and therefore a subset of ESX-1 substrates, seems to be essential for the maintenance of a stable granuloma.

## Discussion

A number of studies have shown that the mycobacterial ESX-1 system plays a pivotal role in mycobacterial pathogenesis [6,21,27,33]. The system affects virulence through secretion of protein effectors with host-modulatory effects. Here, we show that EccA_1_ is not strictly required for the secretion of ESX-1 substrates. The finding that EccA_1_ is important for secretion is in line with previous reports [8,34], but the fact that the role of EccA_1_ is depending on the growth medium is entirely surprising. This difference could also explain the variable results described for the role of EccA_1_ in EsxA secretion by *M*. *tuberculosis* [49]. Of all ESX-1 substrates, EspE, EspF, EspJ and EspK secretion was mostly affected in our *eccA*_*1*_ mutant strain, while secretion of EspB, EsxA/EsxB and PE/PPE was hardly altered. An interesting observation here is the discrepancy between the active secretion of EsxA in the Δ*eccA*_*1*_ strain and at the same time loss of hemolytic activity. Although this observation has been described before, this was always linked to a reduced secretion of EsxA in these strains [8,34]. In a recent study, the importance of EsxA in lysing membranes was questioned [50]. Our results also supports an alternative mechanism: we find a strong correlation between ESX-1 functionality and hemolysis, but this correlation is not seen for EsxA secretion. Our finding is in line with several other recent studies, who showed that both EsxA and the cell-surface lipid PDIM are important for phagosomal rupture and escape by *M*. *tuberculosis* [51–53]. We propose that it is not the loss of secreted EsxA, but the loss of (multiple) surface-exposed Esp proteins that results in hemolytic deficiency.

Even though the Δ*eccA*_*1*_ strain lost its ability to induce membrane lysis, virulence in isolated phagocytes and in zebrafish larvae was only mildly affected in our study. This is in contrast with other studies, who described an attenuated phenotype for similar mutants in *M*. *tuberculosis* and *M*. *marinum* in murine macrophages and zebrafish [8,34]. The latter observations were made after a longer incubation time, which might explain the discrepancy with our study. Distinct phenotypes of the *eccA*_*1*_ mutant in different host cells have also been reported in a genome-wide transposon mutagenesis study [47]. Here, transposon insertions in *M*. *marinum* E11 *eccA*_*1*_ led to severe attenuation in mammalian phagocytic cells but these mutants were hypervirulent in protozoan cells [47]. This suggests that *M*. *marinum* can employ host-specific virulence mechanisms to adapt to different intracellular environments and that EccA_1_ might be essential for secretion and virulence under specific circumstances or in a subset of specific hosts.

The role of EspG as a specific chaperone for the recognition and secretion of cognate PE/PPE proteins has been well established in *M*. *marinum* [30,31]. Our extracellular proteomic study not only confirms the loss of PE/PPE substrate secretion in the *M*. *marinum* Δ*espG*_*1*_ strain, but also reveals the secretion block of other ESX-1 dependent substrates, including EsxA/EsxB. This effect on EsxA/EsxB secretion however was not observed in an *M*. *tuberculosis espG*_*1*_ knock-out strain [33]. EspG proteins bind specifically to the extended helices of the PPE protein, which are absent in Esx proteins. Therefore, the strong effect of *espG*_*1*_ deletion on Esx (and also Esp) protein secretion in *M*. *marinum* is likely indirect due to a mutual dependency in secretion among the ESX-1 substrates [27,31,35]. This co-dependency of PE/PPE and other ESX-1 substrates for secretion is possibly less strict in *M*. *tuberculosis*, explaining that mutating *espG*_*1*_ had no effect on EsxA/EsxB secretion in this species. Because of the severe secretion defect of all detectable ESX-1 substrates, the *M*. *marinum espG*_*1*_ mutant is non-hemolytic and strongly attenuated in macrophage and amoeba, which is in good agreement with previous reports [8]. Furthermore, the loss of *espG*_*1*_ resulted in a strong attenuation in zebrafish, to the same extend as the *eccCb*_*1*_ mutant.

Our most significant and surprising results were obtained for EspH. EspH is specific for the ESX-1 secretion system and is highly conserved among pathogenic mycobacterial species, including *M*. *tuberculosis* and *M*. *leprae*. The latter species has been streamlined into a minimal genome by a process of extensive genome decay. In our study, deletion of this gene abolishes the expression and secretion of two specific ESX-1 substrates EspE and EspF. Furthermore, we could show that EspH specifically interacts with EspE in the cytosol, indicative of chaperone activity. However, the Phyre2 structural prediction program [43] indicated that EspH is shares similarity to YbaB. The first structural study on YbaB strongly indicated that this protein binds DNA as a dimer [44]. However, recent studies indicated that the function of YbaB might be more diverse. One study showed that YbaB is associated with and a target of ClpYQ proteases in *E*. *coli* [54], while another study indicated that overexpression of YbaB enhanced the production of heterologous membrane proteins [55]. Based on the direct interaction of EspH with EspE and that the EspH-like protein EspD stabilizes intracellular EspA/EspC substrates [35], we propose that these YbaB-like proteins encoded by the esx-1 cluster of pathogenic mycobacteria function as dedicated chaperones for specific ESX-1 substrates. Recently, a study of *M*. *tuberculosis* EspL also predicted a high resemblance to YbaB [56], making it tempting to speculate that EspL may as well function as a dedicated chaperone, for instance the ESX-1 substrates encoded by the adjacent genes EspK or EspB. It becomes clear that multiple chaperones, such as EspG_1_, EspD and EspH, are responsible for stabilizing their cognate substrates PE35/PPE68, EspC/EspA and EspF/EspE, respectively. Interestingly, secretion of other substrates of the ESX-1 system, such as EspB, EspK and EspJ, did not seem to be affected by disruption of the *espH* gene. A similar phenotype was observed previously in an *espA*::tn mutant of *M*. *tuberculosis* [26], where secretion of EsxA/EsxB but not EspB was aborted. These results show that interdependence in ESX-1 secretion is not a general feature. Deletion of *espH* did result in reduced secretion of EsxA/EsxB, which was not due to differences in mRNA levels. This hints towards a possible regulation mechanism between the secretion of the central components EsxA/EsxB and the individual Esp substrate (pairs) but not among the Esp proteins themselves.

The *espH* mutant strain showed a loss of hemolytic activity and a reduction of intracellular growth in phagocytic host cells in our study. Strikingly, zebrafish larvae were heavily infected with this mutant strain and showed even hypervirulence at later time points, even though EsxA/EsxB secretion was reduced in this mutant. More detailed analysis showed that initial phagocytosis and primary establishment of an early granuloma was not affected in this mutant. Eventually, a stable cluster of immune cells could not be maintained in larvae infected with the *espH* mutant, with subsequent extracellular bacterial outgrowth and apparent phagocyte death. The discrepancy between *in vitro* and *in vivo* results indicate an essential role for a, yet unknown, host factor involved. It is tempting to speculate that EspE/EspF, the two proteins that are most severely affected by the *espH* deletion, interact with this host factor in order to induce the homeostatic balance between host and pathogen in developing granulomas. Furthermore, because EsxA and EsxB secretion was diminished, other ESX-1 substrates in addition to these central substrates might be involved in the infection process. A candidate might be EspB, whose secretion was not affected in *espH* mutant strain, and was shown to be able to facilitate *M*. *tuberculosis* virulence *in vitro* and *in vivo* in an EsxA-independent way [26].

In summary, this study highlights the complexity of the ESX-1 secretion machinery. We unravel valuable information about the functions of the individual ESX-1 components EccA_1_, EspG_1_ and EspH, all having their unique role in secretion of the different substrate classes. We can conclude that ESX-1 has several different sets of substrates that are involved in distinctive processes required for virulence.

## Materials and methods

### Bacterial strains and cell cultures

All *M*. *marinum* strains used in this study were derived from the wild-type strain M^USA^ [57]. The *eccCb*_*1*_ (M^VU^) strain was previously identified as an ESX-1 secretion mutant with a spontaneous out of frame mutation in *eccCb*_*1*_ [36] and also the knock-out strain *espG*_*1*_ was described before [31]. The knockout strains of *eccA*_*1*_ and *espH* were generated using the mycobacteriophage approach (see below). All strains were routinely cultured on Middlebrook 7H10 plates or in Middlebrook 7H9 medium (Difco) containing ADC supplement or on Sauton medium [58] supplemented with 2% glycerol and 0.015% Tween-80. When required, 0.05% Tween-80 and the appropriate antibiotics were added (25 μg/ml kanamycin (Sigma) and/ or 50 μg/ml hygromycin (Roche). *M*. *marinum* cultures and plates were incubated at 30°C. *E*. *coli* TOP10F’ was used for cloning experiments to generate the complemented plasmids and was grown at 37^°^C on LB plates and in LB medium. Different antibiotics were added to the cultures or plates when necessary at similar concentrations as for *M*. *marinum* cultures.

### DNA manipulation and plasmid construction

All DNA manipulation procedures followed standard molecular biology protocols. Primers were synthesized and purified by Sigma. Phusion polymerase, restriction enzymes and T4 DNA ligase were obtained from New England Biolabs (NEB). Macrogen performed DNA sequencing.

### RNA extraction and RT-PCR analysis

Bacterial RNA was extracted from various *M*. *marinum* strains as described previously [31] and cDNA was synthesized using SuperScript VILO cDNA Synthesis kit (Thermoscientific) according to manufacturer protocol. For the PCR mix the SYBR GreenER qPCR SuperMix (Thermoscientific) was used according to manufacturer instructions, including the addition of ROX dye reference. qRT-PCR was performed in Applied Biosystems 7500 Fast system. The primer sequences used for qRT-PCR are listed in S3 Table. Controls without reverse transcriptase were done on each RNA sample to rule out DNA contamination. The *sigA* gene was used as an internal control.

### Generation of the knockout strains

An *eccA*_*1*_ and *espH* knockout was produced in *M*. *marinum* M^USA^ by allelic exchange using a specialized transducing mycobacteriophage as previously described [59]. High phage titers were prepared following the previously described protocol [31]. Subsequently, the *M*. *marinum* wild-type strain was incubated with the corresponding phage to create *eccA*_*1*_ and *espH* knockouts. Colonies that had deleted the endogenous *eccA*_*1*_ and *espH* were selected on hygromycin plates and tested for sucrose sensitivity, induced by the presence of the *sacB* gene. The deletion was confirmed by PCR analysis and sequencing. To remove the resistance and *sacB* gene, a temperature sensitive phage encoding the γδ-resolvase (TnpR) (a kind gift from Apoorva Bhatt, University of Birmingham, UK) was used, generating an unmarked deletion mutation.

### *M*. *marinum* secretion analysis and immunoblotting

*M*. *marinum* cultures were grown in 7H9 medium supplemented with ADC and 0.05% Tween 80 to mid-logarithmic phase. Bacteria were washed two times and set to OD600 of 0.35 in 7H9 medium containing 0.2% glycerol, 0.2% dextrose and 0.05% Tween 80 for overnight growth. Supernatants were filtered using 0.2 μm filter, concentrated by trichloric acid (TCA) precipitation, washed with acetone and the supernatant pellets were resuspended in solubilisation/denaturation (S/D) buffer (containing 100mM DTT and 2% SDS). Bacteria were washed once with PBS. Aliquots were taken for the whole cell lysate preparations and for Genapol X-080 extraction of cell-surface-attached proteins. Bacteria were incubated with 0.5% Genapol X-080 in PBS for 30 minutes with head-over-head rotation at room temperature. Genapol extracted supernatants were denatured in S/D buffer. The bacterial pellet and Genapol extracted cells were lysed by bead beating for 1 minute two times after which S/D buffer was added. All samples were boiled for 10 minutes at 95^°^C before loading on SDS-PAGE.

### Pulldown assays

For His-tag pulldown, mycobacterial cultures grown to an OD600 of 1.0 were incubated for 1 h with 100 g/ml ciprofloxacin (Sigma), pelleted, washed twice with PBS, and subsequently resuspended in PBS supplemented with Complete protease inhibitor mixture (Roche Applied Science), 1 mM EDTA, and 10 mM imidazole. Cells were broken by two-times passage through a One-Shot cell disrupter (Constant Systems) at 0.83 kbar. Unbroken cells were spun down by repeated centrifugation at 3000 *g*, and subsequently the cell envelope and soluble fractions were separated by ultracentrifugation at 100,000 *g* for 1hr. Membrane-cleared lysates of *M*. *marinum* expressing proteins of interest were incubated with Ni-NTA agarose beads (Qiagen) for 1 h at room temperature with head-over-head rotation. After washing the beads five times with phosphate buffer containing 50 mM NaH_2_PO_4_ and 300 mM NaCl, (pH 8.0), supplemented with 20 mM imidazole, bound proteins were eluted three times by incubation with phosphate buffer containing 400 mM imidazole. Immunoprecipitation of strep-tagged proteins was performed using the Strep-Tactin Sepharose kit (IBA), following the manufacturers protocol.

### SDS-PAGE, immunoblotting, and sera

Proteins were separated by SDS-PAGE and stained with Coomassie Brilliant Blue G-250 (CBB; Bio-Rad), or transferred to nitrocellulose membranes by Western blotting. The membranes were then incubated with different antibodies followed by enhanced chemiluminescence. Primary antibodies used in this study include: anti- GroEL2 (CS44, Colorado state university), anti-PE_PGRS antibody (7C4.1F7) [36], anti-EsxA (Hyb76-8) [60], polyclonal anti-EspE and anti-PPE68 [61,62].

### LC-MS/MS

To investigate the cell-surface attached proteome, samples for LC-MS/MS analysis were prepared using the mild detergent Genapol X-080 as previously described [63]. To prepare the secreted material, the *M*. *marinum* M^USA^ wild-type and the studied ESX-1 mutant and complemented strains were grown to stationary phase in 7H9 medium supplemented with ADC, 0.2% glycerol and 0.05% Tween 80. The supernatant fractions containing secreted proteins were collected and spun at 2500 × *g* for an additional 20 min at 4°C and subsequently filtered through a 0.2 μm pore size membrane to remove residual cells and cell debris. The filtered supernatants were 20 times concentrated using Amicon Ultra-15 Centrifugal 3 kDa molecular weight cut off membrane at 4°C. The retained proteins were TCA precipitated, pelleted, washed in acetone, dried and resuspended in S/D sample buffer to the corresponding OD of 200 units/ml. All samples were analyzed with SDS-PAGE and CBB staining. Total protein lanes of cell surface and culture supernatant proteins were excised in 3 or 1 fragment(s) per lane, respectively, and analyzed by LC-MS/MS as described before [63]. The mass spectrometry proteomics data have been deposited to the ProteomeXchange Consortium via the PRIDE partner repository with the dataset identifier PXD008905.

### Hemolysis assay

*M*. *marinum* strains were grown in 7H9 medium supplemented with ADC and 0.05% Tween-80 till the mid-logarithmic phase. All strains were washed once with PBS and inoculated in 7H9 medium with or without Tween-80 at 0.35 OD600/ml and inoculated for 20 hours. Bacteria were collected by centrifugation, washed once in PBS and diluted in fresh DMEM medium without phenol red (Gibco, Life technologies). Bacteria were quantified by absorbance measurement at OD600 with an estimation of 2.5*10^8^ bacteria in 1 ml of 1.0 OD_600_. At the same time, defibrinated sheep erythrocytes (Oxoid-Thermo Fisher, the Netherlands) were washed five times and diluted in the same fresh DMEM medium. 4.2*10^7^ erythrocytes and 1*10^8^ bacteria were added for one reaction of 100 μl in a round-bottom 96 well-plate, gently centrifuged for 5 minutes and incubated at 32^°^C. After an incubation of 3 hours, cells were resuspended, centrifuged and 80 μl of supernatants were transferred to a flat-bottom 96-wells plate and measured at an absorbance of 405nm to quantify hemoglobin release.

### Host cell growth conditions

The mouse macrophage line RAW264.7 (American Type Culture Collection) was cultured in RPMI 1640 with Glutamax-1 (Gibco) supplemented with 10% fetal bovine serum (FBS; Gibco), 100 U of penicillin/ml, 100 μg of streptomycin/ml at 37°C, 5% CO_2_. A total of 3 × 10^7^ cells was seeded in T175 flasks (Corning). *Acanthamoeba* c*astellanii* was seeded in T175 flasks and grown in PYG medium, which is 0.4M MgSO_4_.7H_2_O, 0.05M CaCl_2_, 0,1 M Sodium citrate.2H_2_O, 0.05M Fe(NH_4_)2(SO_4_)_2_. 6H_2_O, 0.25M Na_2_HPO_4_.7H_2_O, 0.25M KH_2_PO_4_ in distilled water with 2% proteose peptone (W/V, BD 211684) and 0.01% yeast extract. After pH adjustment to 6.5, 2M glucose was added.

### Host cell infection procedure

All bacterial strains were grown until the exponential growth phase, washed with 0.05% Tween 80, spun down and resuspended in RPMI medium. RAW macrophages were infected with a MOI of 5 for 3 hours and incubated at 30°C, 5% CO_2._ Cells were washed in RPMI to remove extracellular mycobacteria and either analyzed immediately or incubated for another 21 hours at 30°C, 5% CO_2._ *A*. *castellanii* (ATCC 30234) infection was performed with a MOI of 1, 3, 9, 27, 54, and 108 to determine optimum MOI, for the remaining experiments MOI 3 was chosen. Incubation for 3 hours or 24 hours was done at 30°C, 5% CO_2._

### Flow cytometry

Uptake of strains in host cells was quantified for all cell lines with a BD Accuri C6 flow cytometer (BD Biosciences) with a 488-nm laser and 585/40-nm filter to detect mEos3.1. A minimum of 5000-gated events was collected per sample per time point, percentage of living cells, percentage of infected cells and median fluorescent intensity per cell was analyzed using BD CFlow software.

### Injection stocks for zebrafish infection

Injection stocks were prepared by growing bacteria until the logarithmic phase (OD_600_ of 0.7–1). Bacteria were spun down at low speed for 1 min to remove the largest clumps, washed with 0.3% Tween-80 in phosphate buffered saline (PBS) and sonicated shortly for declumping. Bacteria were than resuspended in PBS with 20% glycerol and 2% PVP and stored at −80°C. Before use, bacteria were resuspended in PBS containing 0.17% (V/V) phenol red (Sigma) to aid visualization of the injection process.

### Zebrafish infection procedure

Transparent casper zebrafish larvae [64] were removed from their chorion with tweezers and infected at 1 day post fertilization (dpf) via the caudal vein with bacterial suspension containing 50–200 CFU. Injection was performed as described previously [65]. To determine the exact number of bacteria injected, the injection volume was plated on 7H10 plates containing the proper antibiotic selection. At 4 days post infection (dpi) larvae were analyzed with a Leica MZ16FA fluorescence microscope. Bright field and fluorescence images were generated with a Leica DFC420C camera. Infection levels were quantified with a custom-made fluorescent pixel counting software. The software is in house developed and freely available under MIT license. Following analysis, larvae were fixed overnight in 0.4% (V/V) paraformaldehyde (EMS, 100122) in PBS, washed and stored in PBS for immunohistochemistry.

### Ethics statement

All procedures involving *Danio rerio* (zebrafish) larvae were performed in compliance with local animal welfare laws and maintained according to standard protocols (zfin.org). The breeding of adult fish was approved by the institutional animal welfare committee (Animal Experimental licensing Committee, DEC) of the VU University medical center. All protocols adhered to the international guidelines specified by the EU Animal Protection Directive 86/609/EEC, which allows zebrafish larvae to be used up to the moment of free-living (approximately 5–7 days after fertilization). In the current study, zebrafish larvae were used between 1 and 5 days post fertilization.

### Immunohistochemical stain

Larvae were rinsed with 1% PBTx, (1% Triton X-100 in PBS), permeated in 0.24% trypsin in PBS and blocked for 3 hours in block buffer (10% normal goat serum (NGS) in 1% PBTx). Samples were incubated with anti-L-plastin [1:500 (V/V) dilution] in antibody buffer (PBTx containing 1% (V/V) NGS and 1% (W/V) BSA) overnight at RT. Samples were washed with PBTx, incubated for 1 hour in block buffer and stained with an Alexa-Fluor-647 goat-anti-rabbit antibody (Invitrogen A21070, 1:400), overnight at 4°C.

### Confocal microscopy

Confocal analysis was performed on larvae, embedded in 1% low melting-point agarose (Boehringer Mannheim, 12841221–01) in an 8-well microscopy μ-slide (ibidi), Analysis was performed with a confocal laser scanning microscope (Leica TCS SP8 X Confocal Microscope). Leica Application Suite X software and ImageJ software were used to adjust brightness and contrast and to create overlay images and 3D models.

### Graphs and statistical analysis

Graphs were made using Graph Pad Prism 6.0. Pixel counts were logarithmic transformed; error bars represent mean and standard error of the mean. A one-way ANOVA was performed followed by a Bonferroni’s multiple comparison test to analyze statistical significance. Graphs with results of RAW264.7 and *A*. *castellanii* infection experiments show percentage-infected cells of total cells; error bars represent mean and standard error of the mean. Data representing the fold change between 3 and 24 hpi was logarithmic transformed. A two-way ANOVA followed by a Sidak’s multiple comparison test was performed for statistical significance.

## Supporting information

S1 Fig ESX-1 mutant strains show similar growth phenotype and no polar effects caused by the gene deletion. **A.** The deletion of each ESX-1 component had no effect on the growth of the mutant strains. The WT *M*. *marinum* and studied ESX-1 knockout strains were grown in 7H9 medium supplemented with ADC and 0.05% Tween 80. The optical densities of the cultures were measured at a wavelength 600nm. Each color denotes each strain. **B.** No polar effects caused by the deletion of each ESX-1 component to its adjacent genes. Total RNA was isolated from WT *M*. *marinum* M^USA^ and the studied ESX-1 mutant strains. Specific primer sets were used to amplified *espG*_*1*_, *espH and eccA*_*1*_ cDNA. Ct values were normalized for Ct values of the household gene *sigA* and compared to Ct values of the examined genes obtained from WT M^USA^.
(TIF)

S2 Fig Complementation of the mutants restored ESX-1 secretion. The heat map showing that the secretion defects in the Δ*espG*_*1*_, Δ*espH* and Δ*eccA*_*1*_ mutants were restored by overexpressing the complementing plasmid pMV361::*espF-eccA*_*1*_. **A.** Genapol-enriched fractions. **B.** Supernatant fractions.
(TIF)

S3 Fig Deletion of *espH* had no effect at the transcription level but protein level of *espE*. **A.** The deletion of *espH* had no effect on the transcription levels of *espE*, *espF* and *esxA*. Total RNA was isolated from WT *M*. *marinum* M^USA^, *eccCb*_*1*_ mutant strain and the *ΔespH* strain. Specific primer sets were used to amplified *espF* and *espE* cDNA. Also, three different sets of primers of esxA, including *esxA_1*, *esxA_2* and *esxA_3*, were used for *esxA* cDNA. Ct values were normalized for Ct values of the household gene *sigA* and compared to Ct values of the examined genes obtained from WT M^USA^. **B.** The C-terminally Strep-tagged EspE was secreted in the WT M^USA^ and the *ΔespH* complemented strain. Immunoblots of whole cells treated with Genapol (Genapol pellet) and 2-fold excess of Genapol supernatant from *M*. *marinum* WT strain M^USA^, the *eccCb*_*1*_ mutant, the Δ*espH* mutant and the Δ*espH* complemented with the *pMV361*::*espG*_*1*_*-eccA*_*1*_ in which *espH* was C-terminally labeled with a 6xHis tag, all expressing EspE-Strep/EspF, were probed with antibodies against Strep, PE_PGRS and the lysis control GroEL2. **C.** EspE and EspH are soluble in the *M*. *marinum eccCb*_*1*_ mutant. Immunoblot analysis of total (T), soluble (S), and cell envelope (CE) fractions of the *eccCb*_*1*_ mut expressing EspH-His and EspE-Strep/EspF. EspH was detected using mAb directed against the His6 epitope, and EspE was checked using antibodies against both Strep and EspE.
(TIF)

S4 Fig Summary of the ESX-1 secreted proteins discussed in this paper. EsxB, PE35, EspC, EspF, EspJ and EspB, coloured in orange, carry the secretion motif YxxxD/E indicated in red triangle. The WxG conserved motif, highlighted by the blue triangle, is present in EsxA, PPE68, EspA, EspE, EspK (in green) and also in EspB. The variable C terminal domains (CTD) of some ESX-1 substrates are illustrated by the grey box.
(TIF)

S1 Table Strains used in this study. (DOCX)

S2 Table Plasmids used in this study. (DOCX)

S3 Table Primers used in this study. (DOCX)
