# Supplementary figures and images for: EspH is a hypervirulence factor for Mycobacterium marinum and essential for the secretion of the ESX-1 substrates EspE and EspF
Source: PLoS Pathog. 2018 Aug 13;14(8):e1007247. doi: 10.1371/journal.ppat.1007247 (PMC6107294; doi:10.1371/journal.ppat.1007247)

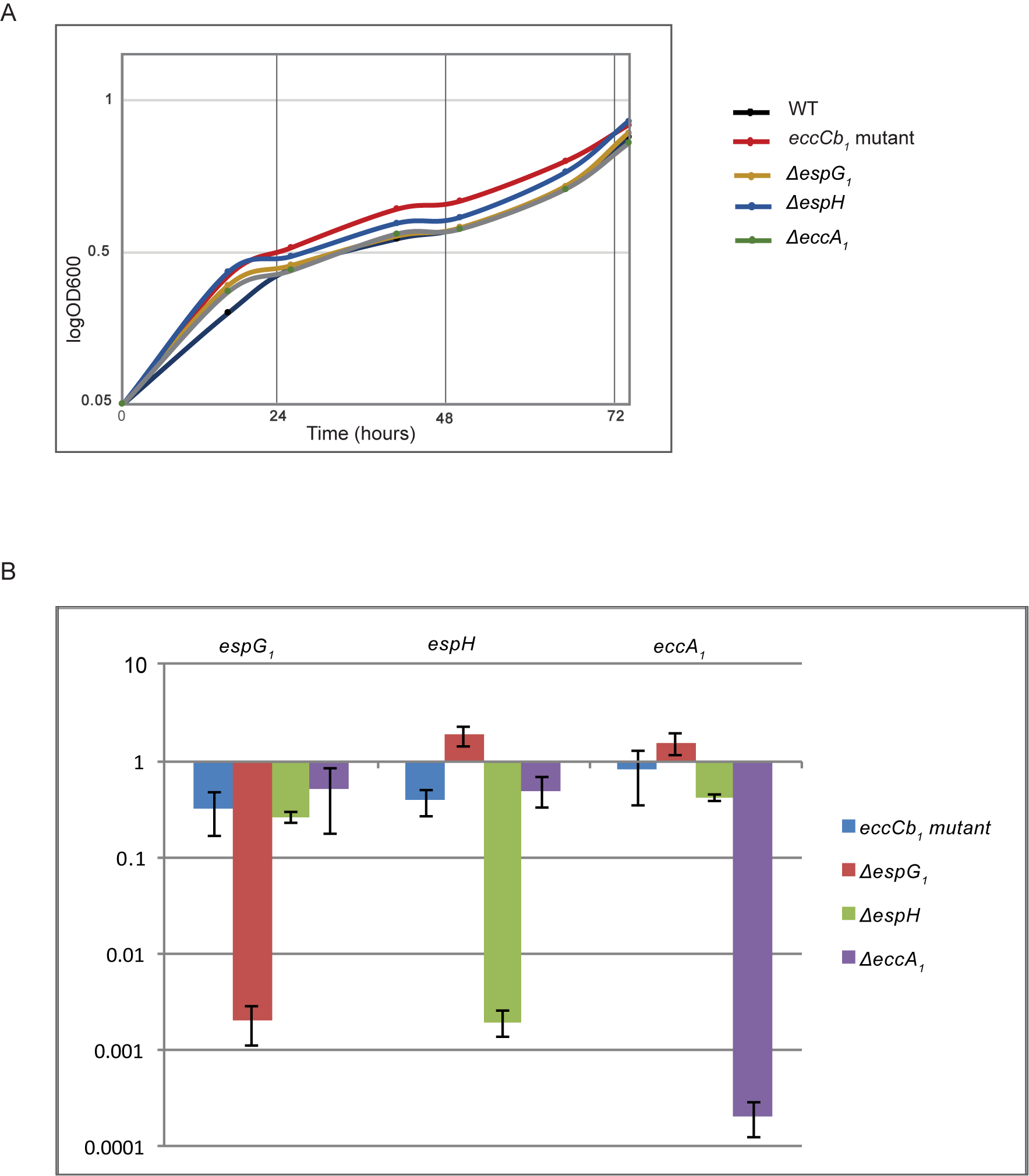

Supplement: S1 Fig — A. The deletion of each ESX-1 component had no effect on the growth of the mutant strains. The WT M. marinum and studied ESX-1 knockout strains were grown in 7H9 medium supplemented with ADC and 0.05% Tween 80. The optical densities of the cultures were measured at a wavelength 600nm. Each color denotes each strain. B. No polar effects caused by the deletion of each ESX-1 component to its adjacent genes. Total RNA was isolated from WT M. marinum MUSA and the studied ESX-1 mutant strains. Specific primer sets were used to amplified espG1, espH and eccA1 cDNA. Ct values were normalized for Ct values of the household gene sigA and compared to Ct values of the examined genes obtained from WT MUSA. (TIF) [file ppat.1007247.s001.tif]

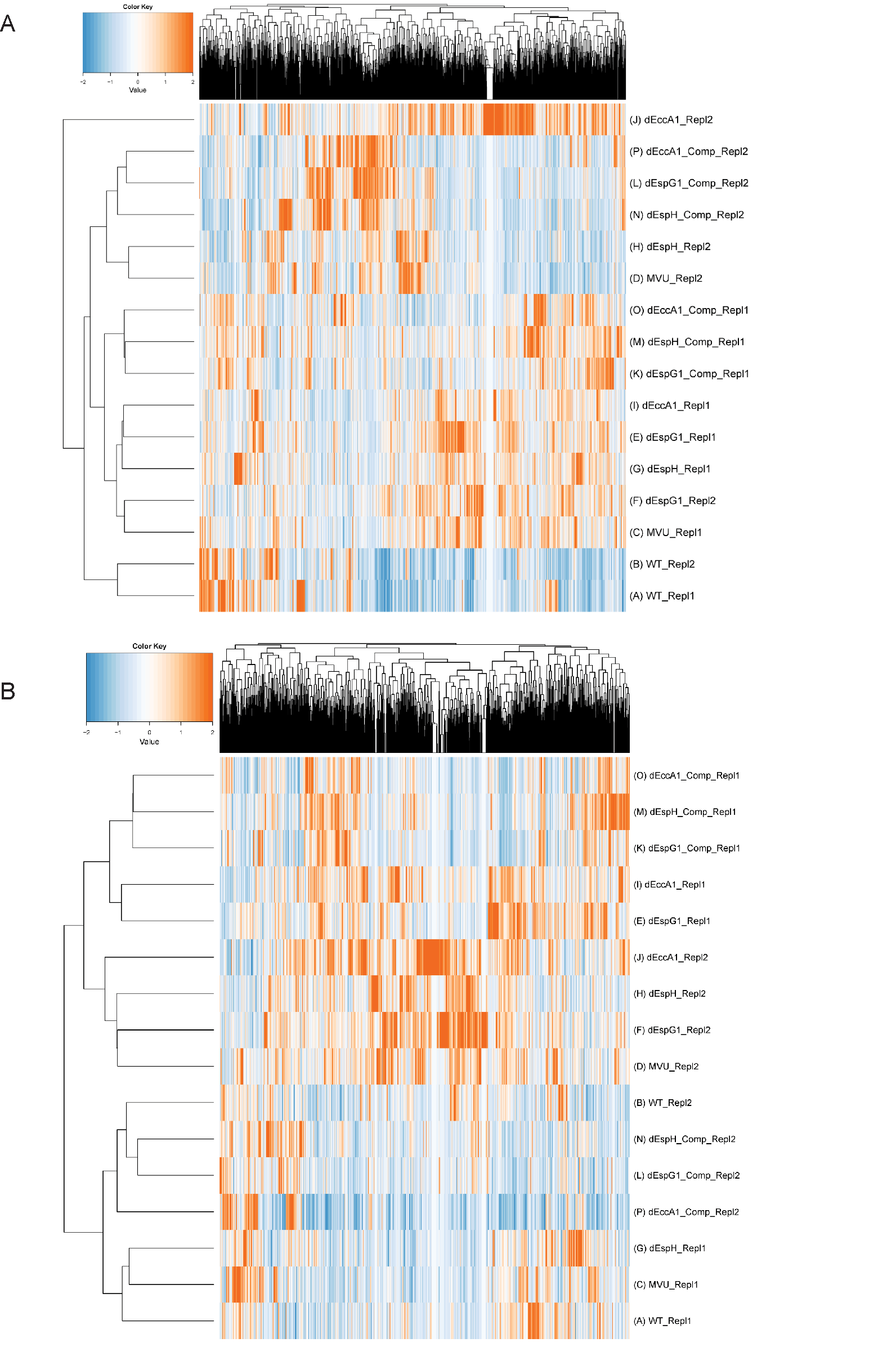

Supplement: S2 Fig — The heat map showing that the secretion defects in the ΔespG1, ΔespH and ΔeccA1 mutants were restored by overexpressing the complementing plasmid pMV361::espF-eccA1. A. Genapol-enriched fractions. B. Supernatant fractions. (TIF) [file ppat.1007247.s002.tif]

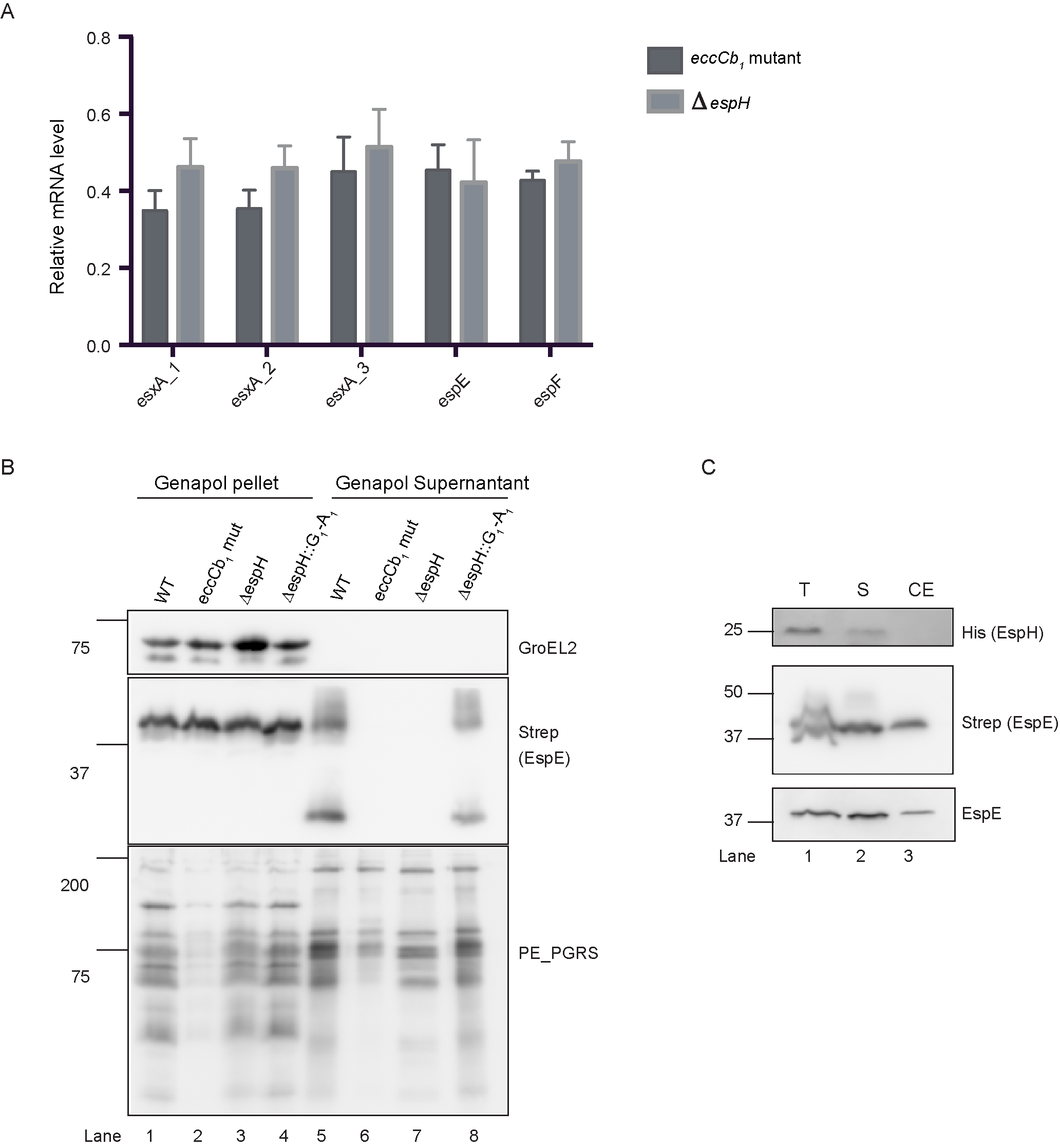

Supplement: S3 Fig — A. The deletion of espH had no effect on the transcription levels of espE, espF and esxA. Total RNA was isolated from WT M. marinum MUSA, eccCb1 mutant strain and the ΔespH strain. Specific primer sets were used to amplified espF and espE cDNA. Also, three different sets of primers of esxA, including esxA_1, esxA_2 and esxA_3, were used for esxA cDNA. Ct values were normalized for Ct values of the household gene sigA and compared to Ct values of the examined genes obtained from WT MUSA. B. The C-terminally Strep-tagged EspE was secreted in the WT MUSA and the ΔespH complemented strain. Immunoblots of whole cells treated with Genapol (Genapol pellet) and 2-fold excess of Genapol supernatant from M. marinum WT strain MUSA, the eccCb1 mutant, the ΔespH mutant and the ΔespH complemented with the pMV361::espG1-eccA1 in which espH was C-terminally labeled with a 6xHis tag, all expressing EspE-Strep/EspF, were probed with antibodies against Strep, PE_PGRS and the lysis control GroEL2. C. EspE and EspH are soluble in the M. marinum eccCb1 mutant. Immunoblot analysis of total (T), soluble (S), and cell envelope (CE) fractions of the eccCb1 mut expressing EspH-His and EspE-Strep/EspF. EspH was detected using mAb directed against the His6 epitope, and EspE was checked using antibodies against both Strep and EspE. (TIF) [file ppat.1007247.s003.tif]

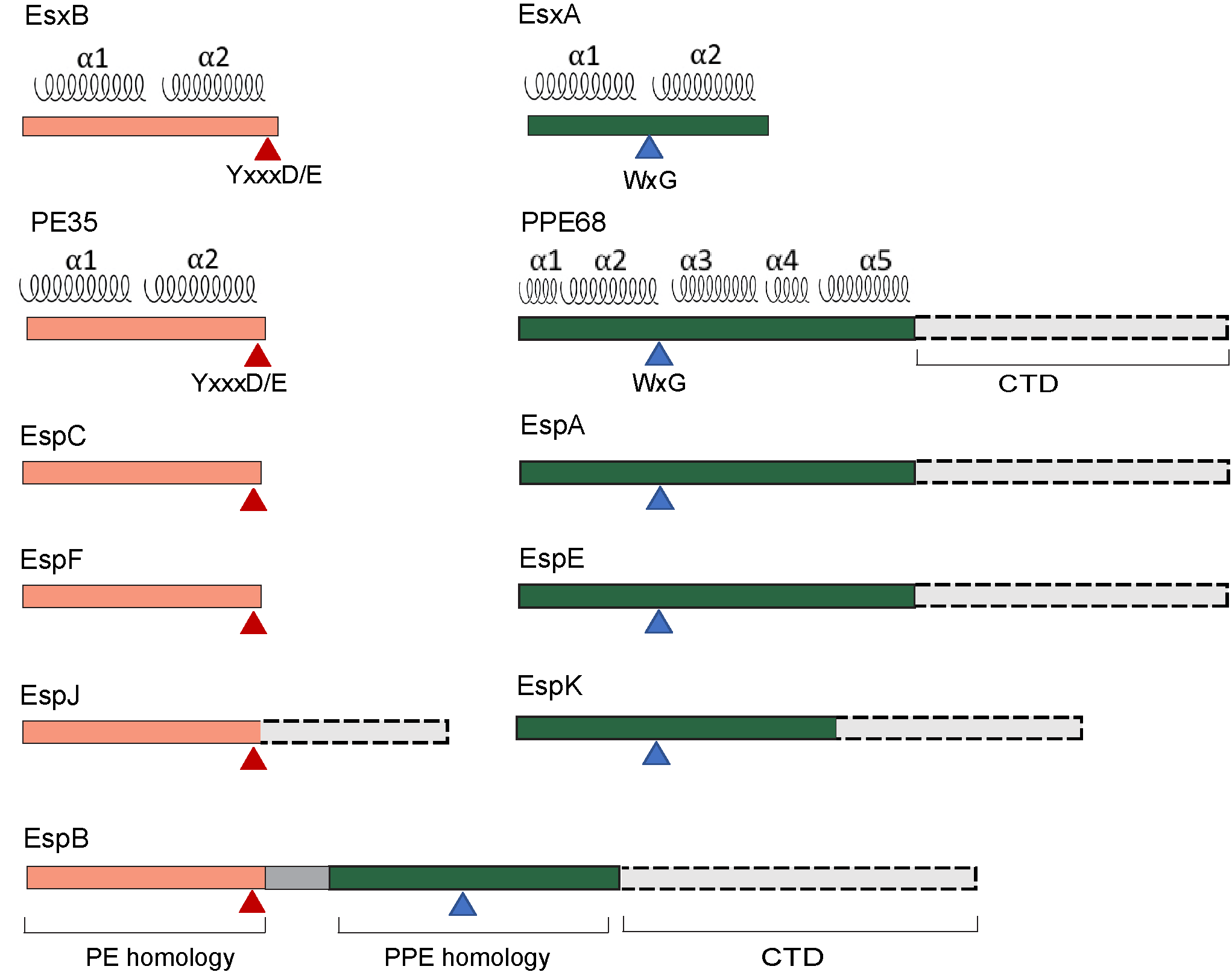

Supplement: S4 Fig — EsxB, PE35, EspC, EspF, EspJ and EspB, coloured in orange, carry the secretion motif YxxxD/E indicated in red triangle. The WxG conserved motif, highlighted by the blue triangle, is present in EsxA, PPE68, EspA, EspE, EspK (in green) and also in EspB. The variable C terminal domains (CTD) of some ESX-1 substrates are illustrated by the grey box. (TIF) [file ppat.1007247.s004.tif]
