# Supplementary material for: EspH is a hypervirulence factor for Mycobacterium marinum and essential for the secretion of the ESX-1 substrates EspE and EspF
Source: PLoS Pathog. 2018 Aug 13;14(8):e1007247. doi: 10.1371/journal.ppat.1007247 (PMC6107294; doi:10.1371/journal.ppat.1007247)
Supplement: S1 Table — (DOCX) [file ppat.1007247.s005.docx]

**S1 Table. Strains used in this study**

| **Strains** | **Characteristics** | **References** |
| --- | --- | --- |
| M^USA^ | WT strain of *M. marinum* | (1) |
| *eccCb_1_* mut (M^VU^) | *M. marnium* MUSA background strain containing the frame-shift mutation in *eccCb1* | (2) |
| *∆espG_1_* | Complete deletion of *espG_1_* in the genome of *M. marinum* MUSA background strain | (3) |
| *∆espH* | Complete deletion of *espH* in the genome of *M. marinum* MUSA background strain | this study |
| *∆eccA_1_* | Complete deletion of *eccA_1_* in the genome of *M. marinum* MUSA background strain | this study |

1. Abdallah AM, Verboom T, Hannes F, Safi M, Strong M, Eisenberg D, et al. A specific secretion system mediates PPE41 transport in pathogenic mycobacteria. Mol Microbiol. 2006;62(3):667–79.

2. Abdallah AM, Verboom T, Weerdenburg EM, Gey Van Pittius NC, Mahasha PW, Jiménez C, et al. PPE and PE-PGRS proteins of Mycobacterium marinum are transported via the type VII secretion system ESX-5. Mol Microbiol. 2009;73(3):329–40.

3. Phan TH, Ummels R, Bitter W, Houben ENG. Identification of a substrate domain that determines system specificity in mycobacterial type VII secretion systems. Sci Rep. 2017;7(February):42704.
