# Supplementary material for: EspH is a hypervirulence factor for Mycobacterium marinum and essential for the secretion of the ESX-1 substrates EspE and EspF
Source: PLoS Pathog. 2018 Aug 13;14(8):e1007247. doi: 10.1371/journal.ppat.1007247 (PMC6107294; doi:10.1371/journal.ppat.1007247)
Supplement: S2 Table — (DOCX) [file ppat.1007247.s006.docx]

**S2 Table. Plasmids used in this study**

| **Plasmids** | **Characteristics** | **References** |
| --- | --- | --- |
| pMV::*espF/espG1/espH/eccA1* | hsp60 promoter, pMV361 backbone plasmid containing a region of *espF/espG_1_/espH/eccA_1_* | Current study |
| pMV::espG1/espH/eccA1 | hsp60 promoter, pMV361 backbone plasmid containing a region of *espG_1_/espH/eccA_1_* | (1) |
| pSMT3::espE-Strep/espF | hsp60 promoter, pSMT3 backbone plasmid containing *espE/espF* in wich *espE* is C-ternimally tagged with Strep | Current study |
| pSMT3::meoS 3.1 | hsp60 promoter, pSMT3 backbone containing mEos3.1 | Van Leeuwen et al – submitted  (2,3) |
| pMV361 empty | hsp60 promoter, pMV361 empty plasmid |  |

1. Phan TH, Ummels R, Bitter W, Houben ENG. Identification of a substrate domain that determines system specificity in mycobacterial type VII secretion systems. Sci Rep. 2017;7(February):42704.

2. Meijer AH, van der Sar AM, Cunha C, Lamers GEM, Laplante M a, Kikuta H, et al. Identification and real-time imaging of a myc-expressing neutrophil population involved in inflammation and mycobacterial granuloma formation in zebrafish. Dev Comp Immunol. 2008 Jan;32(1):36–49.

3. Zhang M, Chang H, Zhang Y, Yu J, Wu L, Ji W, et al. Rational design of true monomeric and bright photoactivatable fluorescent proteins. Nat Methods. 2012;9(7):727–9.
