# Supplementary material for: EspH is a hypervirulence factor for Mycobacterium marinum and essential for the secretion of the ESX-1 substrates EspE and EspF
Source: PLoS Pathog. 2018 Aug 13;14(8):e1007247. doi: 10.1371/journal.ppat.1007247 (PMC6107294; doi:10.1371/journal.ppat.1007247)
Supplement: S3 Table — (DOCX) [file ppat.1007247.s007.docx]

**S3 Table: Primers used in this study**

| **Purposes** | **Primer name** | **Sequence** |
| --- | --- | --- |
| *∆espH* generation | *espH* KO LF | TTTTTTTTCACAAAGTGGCCAAACCCATAGCGAGTAG |
|  | *espH* KO LR | TTTTTTTTCACTTCGTGTGTGGCGTCCCTTTCTGAAC |
|  | *espH* KO RF | TTTTTTTTCACAGAGTGGCGGCCGAAGCCGAGGTATT |
|  | *espH* KO RR | TTTTTTTTCACCTTGTGCTAGTCCGGCGAGCATGTTG |
| *∆eccA_1_* generation | *eccA_1_* KO LF | TTTTTTTTCACAAAGTGACATCCCGCAAGAGGATCTG |
|  | *eccA_1_* KO LR | TTTTTTTTCACTTCGTGGTATCACCGTTCGTTGTAAC |
|  | *eccA_1_* KO RF | TTTTTTTTCACAGAGTGGGAAACCAACGAGGGTCTAC |
|  | *eccA_1_* KO RR | TTTTTTTTCACCTTGTGGCTCCCATTCCCAACACAAG |
| *espG_1_* qPCR | *espG_1_* qPCR FW | AACTGTACGGCAGCTTCCTC |
|  | *espG_1_* qPCR RV | ATTAAGTCAACCTCGGGCGG |
| *espH* qPCR | *espH* qPCR FW | GATGCACTTCACGGGCTGAC |
|  | *espH* qPCR RV | CATGTTCGCAGCCTTGTCGG |
| *eccA_1_* qPCR | *eccA_1_* qPCR FW | TGGCCGAAGCCCAAGAAGAA |
|  | *eccA_1_* qPCR RV | CTGACTGGCCCTCGTACTCG |
| *espF* qPCR | *espF* qPCR FW | GCGGCCGAGATCAGATTGTT |
|  | *espF* qPCR RV | ACCCACGGCTCATTCACCT |
| *espE* qPCR | *espE* qPCR FW | AGGAATCGCCGACAAGATGG |
|  | *espE* qPCR RV | ATCAGGTTGCCGGTCAGATA |
| *esxB* qPCR | *esxB* qPCR FW | ATCTCCGGTGACCTGAAGAC |
|  | *esxB* qPCR RV | TTCGGCCTTCTGCTTGTTGG |
| *esxA* qPCR | *esxA* qPCR FW | GGCAGCATCCAGCGCAATTC |
|  | *esxA* qPCR RV | AGCTTGTGCAGCGACTGCTT |
| sigA qPCR | *sigA_*FW | TCGAGGTGATCAACAAGCTG |
|  | *sigA_*RV | ATTTCTTTGGCCAGCTCCTC |
| pMV::  *espF/espG_1_*  */espH/eccA_1_* | F_PacI_  espF | TCTCTTAATTAACGGCTCACTGGCCTACCAAA |
|  | R_EccA1_  HindIII | GGGGGGAAGCTTTCACTCTCTCATATTGAGGTGTG |
| pMV::  *espG_1_*  */espH.His*  */eccA_1_* | Fw_PacI_  EspG1 | GGGGGGTTAATTAAATGACCGGTCCGCTCG |
|  | Rv_EspH_  His | TCAATGGTGGTGGTGATGATGCCGTTCGTTGTAACGAGAGGTG |
|  | Rv_ EccA1_  HindIII | GGGGGG*AAGCTT*TCACTCTCTCATATTGAGGTGTG |
|  | Fw_His tag_EccA1 | CATCATCACCACCACCATTGATACATGACTGATCGCCTGGCC |
| pSMT3::  *espE.Strep*  */espF* | EspF Fw | GAGGAAAGGTCTACCCCCATGTATCCGTATGATGTTCCTGATTATGCT ACAGGACTACTGAACGTCGTG |
|  | EspF_Rv | AGCATAATCAGGAACATCATACGGATACATGGGGGTAGACCTTTCCTC |
|  | espE_strep Rv | CTACTTCTCGAACTGCGGATGCGACCAGAGGAGGGTCCCCTCG |
|  | Strep_espF Fw | CGAGGGGACCCTCCTCTGGTCGCATCCGCAGTTCGAGAAGTAGTCCGGGCAACCG |
|  | Fw EspE NheI | CCCCCGCTAGCATG*GTGCCAAAGGGAAG* |
